# Beyond Potency: Emerging Determinants and Optimization Strategies Enhancing Therapeutic Efficacy of Adult Stem Cells

**DOI:** 10.1002/advs.76207

**Published:** 2026-06-19

**Authors:** Soo‐Rim Kim, Yun Jae Jung, Hwa‐Yong Lee

**Affiliations:** ^1^ Department of Health Sciences and Technology GAIHST Gachon University Incheon Republic of Korea; ^2^ Department of Molecular Medicine School of Medicine Gachon University Incheon Republic of Korea; ^3^ Department of Microbiology College of Medicine Gachon University Incheon Republic of Korea; ^4^ Division of Science Education Kangwon National University Chuncheon Republic of Korea

**Keywords:** 3D Culture, adult stem cells, intrinsic enhancement, microenvironment, physical stimulation, pluripotency, therapeutic efficacy

## Abstract

Adult stem cell therapies are advancing, yet inconsistent clinical benefit indicates that in vitro potency only partially predicts in vivo performance. Framing efficacy through a systems lens, this review integrates three interlocking levers: (i) cell‐intrinsic programs (tissue imprint, transcriptional/epigenetic state, senescence and metabolic fitness), (ii) microenvironmental constraints and designable cues, and (iii) pre‐ and post‐delivery engineering interventions. We highlight how spatially organized biofabrication—especially 3D bioprinting that tunes geometry, stiffness, and factor presentation—converts a simple cell infusion into an architected niche that stabilizes survival, aligns angiogenesis, and accelerates functional integration. We also synthesize optimization levers spanning biochemical/cytokine licensing, hypoxic and mechanical conditioning, genetic/epigenetic programming, and extracellular‐vesicle–centered strategies; exemplar platforms show that 3D culture and printing can amplify small EV yield and pro‐regenerative content, translating to enhanced vascularization and repair in vivo. Translationally, early‐ and mid‐phase studies show adult stem cells —particularly MSCs—are broadly deliverable with acceptable safety; however, heterogeneity in sourcing, manufacture, dose/route, endpoints, and follow‐up undermines comparability, meta‐analysis, and payer confidence. We outline a path toward adequately powered, multicenter randomized trials under harmonized controls and mechanism‐linked potency assays with standardized clinical and biomarker endpoints. Finally, we examine the regulatory architecture: divergent definitions (manipulation, homologous use), GMP/GTP interpretations, and registry opacity enable uneven evidentiary thresholds and, at times, unregulated offerings—conditions that demand convergence on technical standards, explicit oversight transparency, and risk‐based enforcement. Throughout, mesenchymal stem/stromal cells (MSCs) serve as the principal exemplar of adult stem cells, with other adult stem cell populations cited to show that these principles generalize across cell types.

## Graphical abstract

Adult stem cell therapy requires more than high in vitro potency. This review proposes a systems framework in which cell‐intrinsic programs, instructive microenvironmental cues, and pre‐/post‐delivery engineering are co‐designed under standardized translational rules. By integrating source matching, senescence and metabolic fitness, niche biofabrication, priming, spheroids/extracellular vesicles, genetic/epigenetic tuning, and mechanism‐linked potency assays, adult stem cell products can be converted into reproducible and clinically durable therapies.

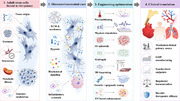

## 1 Introduction

Adult stem cell–based therapies have progressed from proof‐of‐concept studies to a diversified clinical enterprise that now spans autoimmune [1], cardiovascular [2], neurodegenerative [3], musculoskeletal [4], and periodontal [5] indications. Yet, durable benefit remains inconsistent across trials and disease settings, underscoring that in vitro potency alone only partially predicts in vivo performance. Converging evidence situates adult stem cell efficacy within a systems framework: outcomes are jointly determined by (i) cell‐intrinsic programs (tissue of origin [6], transcriptional/epigenetic state [7], senescence burden [8], metabolic fitness [9]), (ii) microenvironmental constraints and cues (matrix composition [10] and mechanics [11], oxygen tension [12], inflammatory tone [13], vascular access [14]), and (iii) engineered interventions that purposefully reshape those variables before—through manufacturing and genetic [15]/epigenetic programming [16]—or after delivery—through biomaterials [17] and device‐enabled [18] niche design. This review synthesizes those determinants and organizes actionable levers that bridge bench potency with clinically meaningful, durable responses. The determinants and intervention strategies introduced here are examined in depth at the opening of their respective sections.

On the cell‐intrinsic axis, source imprints and molecular state strongly condition therapeutic behavior. Even within the broad MSC class, tissue origin (bone marrow, adipose, umbilical cord, dental pulp) confers distinct growth kinetics, lineage bias, and paracrine/immunomodulatory profiles—features that can amplify or blunt efficacy depending on the indication [6, 19]. Beyond source, targeted augmentation of pro‐survival, pro‐migratory, and pro‐regenerative circuits has emerged as a rational strategy: enforced expression of trophic factors and master regulators can harden cells against oxidative/apoptotic stress, enhance reparative secretomes, and improve lesion‐matched homing, while cautionary data remind that indiscriminate activation of survival pathways can accelerate senescence or maladaptive remodeling [20, 21, 22]. In parallel, epigenetic interventions—either pharmacological or via exosome‐mediated delivery—modulate chromatin accessibility and enhancer logic to (re)activate regenerative gene networks, restore anti‐inflammatory and mitochondrial quality‐control programs, and rejuvenate late‐passage or disease‐impaired MSCs [16, 23, 24, 25]. Together, these findings argue for mechanism‐anchored “cell preparation” rather than source‐agnostic procurement.

Equally decisive is the context into which cells are placed. Spatially organized biofabrication and microenvironmental engineering convert adult stem cell therapy from a suspension infusion into an architected, instructive niche. Advances in 3D bioprinting show that precise control of geometry, stiffness, and factor presentation can direct cell fate and function—stabilizing survival, aligning angiogenesis with tissue architecture, and enabling rapid functional integration. Lattice, banded, tubular, and lamellar prints deliver heterotypic interfaces and diffusion‐aware layouts that program vascularization, epithelial–stromal organization, and even directed neural circuit formation, illustrating how “place” and “neighbors” are themselves therapeutics [18, 26, 27, 28, 29]. These orthogonal design knobs—material, mechanics, geometry, and spatially resolved cargo—provide a scalable route to standardize the host context that adult stem cells will encounter.

The translational landscape is advancing but uneven. Across early‐ and mid‐phase clinical studies, adult stem cell products (particularly MSCs) are consistently deliverable with acceptable safety and adherence, and several programs report signal‐level improvements in disease activity or tissue regeneration under rigorous monitoring [30, 31, 32, 33]. Still, the field remains methodologically fragile: many studies are small, single‐center, or single‐arm; sourcing, manufacturing, dose/schedule, and delivery routes vary widely; outcome measures and follow‐up horizons are heterogeneous; and correlative biomarker frameworks (e.g., MRD, imaging, or fluid biomarkers) are often under‐specified. These realities complicate meta‐analysis, payer deliberations, and the development of robust patient‐selection rules—highlighting the need for adequately powered, multicenter randomized trials built on harmonized manufacturing controls, potency assays linked to mechanism, and standardized clinical/biomarker endpoints.

Regulatory architecture and standardization further shape what “optimized” can credibly reach patients. Major jurisdictions classify adult stem cell products within risk‐based medicinal categories and require GxP‐anchored manufacturing, pharmacovigilance, and CTD‐style dossiers, but key definitions (minimal vs. substantial manipulation, homologous use), GMP/GTP interpretations, potency/identity assay expectations, and registry transparency are not fully aligned across borders [34, 35, 36]. Dual‐track systems that encourage early clinical exploration have accelerated pipelines yet exposed gaps in quality systems and data portability from investigator‐initiated studies to registration‐grade evidence. In parallel, the visibility afforded by trial registries can be co‐opted by unregulated offerings, necessitating clearer disclosure of competent‐authority oversight and IRB review and stronger inspection/enforcement. Looking forward, frameworks must also anticipate new modalities—engineered exosomes, organoid‐derived products, and gene‐edited cells—to ensure that ASC therapies advance under harmonized, enforceable, and evidence‐generating rules.

Against this backdrop, our review is structured to: (i) delineate intrinsic determinants of adult stem cell function and how they can be engineered via pro‐survival/pro‐regenerative gene programs; (ii) define microenvironmental and biofabrication strategies—including 3D bioprinting of spatially organized constructs—that convert the host niche into a therapy amplifier; (iii) synthesize epigenetic approaches that reset regenerative networks in diseased or senescent cells; (iv) critically appraise current clinical outcomes and limitations; and (v) map the global regulatory and standardization terrain that governs translation. Collectively, these threads support a unified thesis: durable clinical efficacy is most likely when cells and context are co‐designed—installing the right programs in the right places under the right rules.

A clarification of scope and terminology is warranted given the breadth of this field. Although the determinants and optimization strategies discussed in this review apply to adult stem cells broadly, we use mesenchymal stem/stromal cells (MSCs) as the principal exemplar throughout. MSCs are the most extensively characterized and clinically advanced adult stem cell type, and therefore, provide the richest evidence base from which to distil transferable principles of therapeutic efficacy. Where other adult stem cell populations—including hematopoietic stem cells, skeletal muscle satellite cells, intestinal stem cells, and neural stem/progenitor cells—offer instructive evidence, they are introduced explicitly to demonstrate that a given determinant or engineering principle generalizes beyond MSCs. Accordingly, statements that refer specifically to “MSCs” should be read as MSC‐specific, whereas references to “adult stem cells” denote principles intended to apply across cell types, with MSCs serving as the representative case for clinical translation.

## 2 Key Determinants Governing Therapeutic Efficacy of Adult Stem Cells

In keeping with the scope defined above, the determinants examined in this section are presented as general principles of adult stem cell efficacy, with MSCs serving as the principal exemplar; other adult stem cell populations are cited at specific points where they demonstrate that a determinant operates beyond MSCs

### 2.1 Stem Cell Intrinsic Determinants

On the cell‐intrinsic axis, source imprints and molecular state strongly condition therapeutic behavior. Even within the broad MSC class, tissue origin (bone marrow, adipose, umbilical cord, dental pulp) confers distinct growth kinetics, lineage bias, and paracrine/immunomodulatory profiles—features that can amplify or blunt efficacy depending on the indication [6, 19]. Beyond source, targeted augmentation of pro‐survival, pro‐migratory, and pro‐regenerative circuits has emerged as a rational strategy: enforced expression of trophic factors and master regulators can harden cells against oxidative/apoptotic stress, enhance reparative secretomes, and improve lesion‐matched homing, while cautionary data remind that indiscriminate activation of survival pathways can accelerate senescence or maladaptive remodeling [20, 21, 22]. In parallel, epigenetic interventions—either pharmacological or via exosome‐mediated delivery—modulate chromatin accessibility and enhancer logic to (re)activate regenerative gene networks, restore anti‐inflammatory and mitochondrial quality‐control programs, and rejuvenate late‐passage or disease‐impaired MSCs [16, 23, 24, 25]. Together, these findings argue for mechanism‐anchored “cell preparation” rather than source‐agnostic procurement.

#### 2.1.1 Cell Source and Tissue Origin Specificity

The therapeutic potential and biological functions of MSCs are significantly influenced by their cell source and tissue of origin. Despite sharing common features, MSCs from different tissues exhibit distinct functional characteristics, including proliferation capacity, differentiation potency, lineage specificity, and secretory profiles. Recent studies have highlighted tissue‐dependent variations in MSCs, emphasizing superior osteogenic capacity in bone marrow‐derived MSCs, enhanced adipogenic potential in adipose tissue‐derived MSCs, robust immunomodulation in placental MSCs, higher proliferative activity in Wharton's jelly MSCs, and specialized regenerative functions in perinatal tissue‐derived MSCs. Understanding these inherent differences is crucial for selecting optimal MSC sources tailored to specific therapeutic applications.

A consistent principle emerges across comparative studies of MSC sourcing: the tissue of origin imprints a durable functional bias that is best understood as epigenetic memory rather than transient culture adaptation, making source selection a primary determinant of therapeutic efficacy rather than a logistical convenience. Bone marrow–derived MSCs are reproducibly osteogenically primed, adipose–derived MSCs are biased toward adipogenic and pro–angiogenic programs, placental and perinatal MSCs toward immunomodulation, and Wharton's jelly–derived MSCs toward high proliferative output [19, 37, 38]. Critically, this bias is mechanistically anchored rather than merely descriptive: promoter methylation of lineage–determining transcription factors tracks directly with differentiation outcome—Runx2 is hypomethylated in bone marrow MSCs and PPARγ in adipose MSCs—indicating that tissue–imprinted chromatin states, not surface phenotype, set lineage potential [37]. Single–cell transcriptomic analyses resolve these bulk–level biases into discrete, source–specific subpopulations aligned with vascular, reproductive, cardiogenic, or neurogenic programs, and show that subpopulation composition itself shifts with passage and oxygen tension [6].

Even within ostensibly similar perinatal tissues, functional divergence is pronounced and clinically consequential: amnion–derived MSCs are intrinsically senescence–prone, chorion–derived MSCs are cardiogenically biased, and umbilical cord MSCs favor neural differentiation [38]. Taken together, these data argue against source–agnostic procurement. Because each tissue confers a distinct and partially heritable program, the actionable strategy is indication–matched sourcing—selecting the donor tissue whose imprinted bias aligns with the regenerative demand of the target lesion—while recognizing that source must ultimately be weighed alongside translational variables such as donor age, harvest invasiveness, and manufacturing scalability.

**FIGURE 1:**
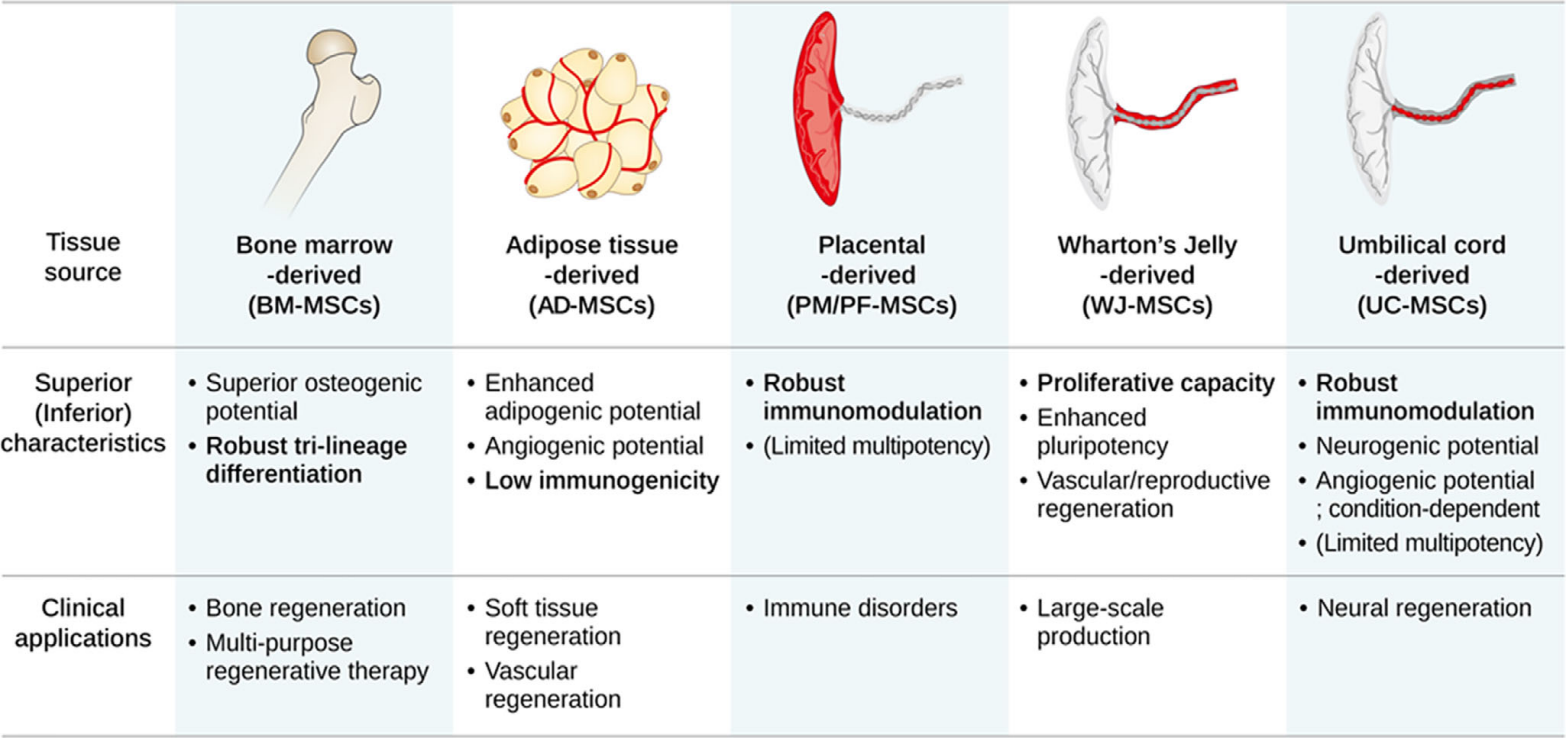
Tissue origin–specific functional heterogeneity of human mesenchymal stromal/stem cells (MSCs) and its therapeutic implications. Schematic summary comparing representative MSC sources—bone marrow (BM‐MSCs), adipose tissue (AD‐MSCs), placenta (maternal/fetal; PM/PF‐MSCs), Wharton's jelly (WJ‐MSCs), and umbilical cord (UC‐MSCs)—highlighting source‐dependent differences in proliferation, lineage bias, immunomodulatory capacity, and regenerative specialization. BM‐MSCs generally show enhanced osteogenic potential and robust tri‐lineage differentiation, whereas AD‐MSCs display strong adipogenic and pro‐angiogenic tendencies with relatively low immunogenicity. Placental/perinatal MSCs exhibit potent immunomodulation, while WJ‐MSCs are characterized by high proliferative activity and broad regenerative utility, supporting scalable manufacturing. UC‐MSCs demonstrate robust immunomodulation with context‐dependent angiogenic and neurogenic features. These functional biases are supported by reported differences in lineage marker expression and staining outcomes, tissue‐imprinted epigenetic memory (e.g., promoter methylation of lineage‐determining transcription factors), and single‐cell transcriptomic subpopulation structures that align with vascular, reproductive, cardiogenic, or neurogenic programs across tissue sources. Original figure created by the authors. Copyright 2026, The Authors.

Beyond the tissue of origin, the practical translatability of a stem cell source is governed by a set of donor‐ and process‐dependent variables that determine whether a biologically promising population can be manufactured into a consistent therapeutic product. Foremost among these is donor age: cells procured from older donors typically show reduced proliferative capacity, attenuated differentiation, and a weaker trophic secretome, reflecting an accumulated senescent burden that constrains both potency and expandability (the senescence mechanisms underlying this age effect are detailed in Section 2.1.3). Compounding age, pronounced donor‐to‐donor variability is observed even within a single tissue type, with potency, immunomodulatory capacity, and expansion kinetics differing markedly between individuals—a heterogeneity that directly undermines lot‐to‐lot comparability and is a recognized contributor to inconsistent clinical outcomes. Source choice is further shaped by harvest invasiveness: bone marrow aspiration is painful and yields relatively few cells, adipose tissue is obtained less invasively and at higher yield, and perinatal tissues such as umbilical cord and Wharton's jelly are recovered non‐invasively from otherwise‐discarded material. These tissue origin–dependent differences in MSC functional properties and their indication–matched therapeutic applications are summarized in Figure 1.

#### 2.1.2 Stemness‐Associated Molecular Signatures

A recurring source of imprecision in this field is the framing of MSC potency in terms of pluripotency. MSCs are multipotent, not pluripotent: although MSC populations express transcription factors classically associated with pluripotency—including SOX2, NANOG, KLF4, MYC and LIN28—their presence does not confer pluripotency and is more accurately interpreted as a stemness program that sustains self‐renewal, proliferative reserve and a differentiation‐poised epigenetic state. The value of these molecular signatures is therefore mechanistic rather than taxonomic: their expression is not a label of cell identity but a quantitative index of where a cell lies on a self‐renewal‐to‐commitment continuum, and it is this position that determines regenerative potency.

It follows from this view that a stemness signature does not describe a population uniformly but stratifies it into a functional hierarchy. Single‐cell profiling of bone marrow and Wharton's jelly MSCs resolves this hierarchy directly, placing a stemness‐high, actively proliferating subpopulation at the apex of a differentiation trajectory that feeds lineage‐primed progenitors [39], and cross‐tissue comparisons show that core stemness regulators are expressed unevenly between sources, with their abundance tracking tri‐lineage differentiation and immunomodulatory potency [40]. The mechanistic implication is concrete: the therapeutic capacity of an MSC preparation is set by the size and fitness of its stemness‐high fraction, not by the average expression of any single marker—which is why bulk‐population phenotyping is often a weak predictor of potency.

Because the stemness signature predicts potency, it becomes therapeutically actionable in two complementary ways: as a prospective selection criterion and as a dynamic quality attribute. For selection, the signature can be reduced to practical handles—surface neuropilin‐2 prospectively identifies MSC clones with superior proliferation, differentiation and migration, and VEGF‐C/NRP2 signaling further amplifies these properties, providing a live‐cell enrichment route [41], while a conserved, cross‐tissue proteostasis‐gene program distinguishes bona fide MSCs and predicts their homeostatic capacity [42]. For monitoring, the same signature is informative precisely because it is environmentally erodible: obesity‐associated metabolic stress downregulates SOX2, KLF4, and TRA‐1‐60 and shifts adipose MSCs toward an inflammatory, senescence‐associated state, demonstrating that the signature reports cell fitness dynamically rather than denoting a fixed identity [43]. The translational synthesis is therefore twofold: stemness‐associated profiles should be used both to enrich potent subpopulations at the point of manufacture and to flag loss of fitness before clinical deployment, so that the signature functions as an operational instrument for cell selection and release rather than as a descriptive marker list.

#### 2.1.3 Cellular Aging and Senescence Status

The therapeutic potential of adult MSCs is profoundly influenced by their cellular aging and senescence status, which dictate self‑renewal, differentiation capacity, and paracrine functionality. As MSCs age, they progressively accumulate senescence‐associated phenotypes, including cell cycle arrest, telomere shortening, DNA damage, oxidative stress, and acquisition of a senescence‐associated secretory phenotype (SASP), which collectively impair regenerative efficacy. Senescent MSCs not only exhibit diminished proliferation and lineage differentiation but can also exert deleterious paracrine effects, promoting local inflammation and tissue degeneration. Conversely, strategies that delay or reverse cellular senescence—through genetic modulation, optimized culture conditions, or mechanical and biochemical interventions—can preserve MSC stemness, enhance immunomodulatory activity, and extend their functional lifespan. Thus, monitoring and mitigating cellular aging is essential for maximizing the therapeutic outcomes of MSC‑based regenerative therapies.

Two mechanistically distinct processes are often conflated under the label of MSC “aging,” and separating them is essential for translation: chronological donor aging, which is intrinsic to the harvested tissue, and replicative or culture–induced senescence, which is acquired during ex vivo expansion. Beyond their different origins, the two processes differ in driver and tempo: donor aging is a cumulative, organism‐level process—reflecting in vivo inflammaging and epigenetic drift—that is already imprinted in the cells at the point of harvest, whereas culture senescence is acquired progressively ex vivo, driven by the oxidative and replicative stress of expansion and accelerating with passage number. Both converge on a common, potency–eroding phenotype—cell–cycle arrest, telomere attrition, oxidative DNA damage and a senescence–associated secretory phenotype (SASP)—but they are addressable by different interventions. Crucially, senescent MSCs are not merely inert: they can become active drivers of tissue degeneration. Intra–articular injection of senescent MSCs alone is sufficient to trigger cartilage breakdown through a pro–degenerative SASP, converting a putative therapeutic into a pathogenic agent [44], and senescence also undermines the immunomodulatory axis by downregulating PD–L1 and abolishing T–cell suppression—a deficit partially rescued by PD–L1 enrichment or by selecting low–senescence perinatal sources [45].

Because senescence is a modifiable state, the more important message is that both the donor–aging and culture–senescence arms can be therapeutically countered. Culture senescence is mitigated by manufacturing choices that limit oxidative burden, such as low–density expansion, which preserves osteogenic potential at late passage by reducing ROS and oxidative DNA damage [46]; donor aging is reversible by targeted conditioning, with physiological mechanical loading reactivating antioxidant and DNA–repair programs to rejuvenate MSCs from aged donors [47], and FOXO3–engineered senescence–resistant progenitors decelerating epigenetic aging clocks across multiple organs in primates [8]. The unifying translational requirement that follows is rigorous senescence surveillance: because senescent cells can actively harm the host, MSC potency should be quantified—through SA–β–gal, p16/p21, telomere, and SASP readouts—as a release criterion before in vivo application, rather than inferred from passage number alone.

#### 2.1.4 Immunogenicity and Immune‐Modulatory Potential

The clinical success of an adult MSC therapy, particularly in the allogeneic setting that makes off‐the‐shelf products feasible, depends not only on regenerative capacity but on how the cells interact with the host immune system. This interaction has two faces—how readily the cells are recognized and cleared, and how effectively they suppress damaging effector responses—and both are shaped by intrinsic properties such as tissue origin, donor sex, and developmental stage, as well as by extrinsic cues such as inflammatory licensing and the engineered microenvironment. Framed this way, immunobiology is not a fixed property of an MSC preparation but a determinant that can be deliberately optimized, and the central question for translation is how its two faces should be balanced and tuned.

These two faces are best treated as mechanistically separable axes: immunogenicity—the likelihood of being recognized and cleared by the host—and immunomodulatory potency—the capacity to actively suppress effector responses. The principle emerging from recent work is that the two are governed by partly independent variables and can therefore be tuned separately, so that an optimal allogeneic product minimizes the first while maximizing the second. Tissue origin illustrates this dissociation directly: adipose‐derived MSCs combine low MHC‐I/II expression with immunosuppressive capacity comparable to that of bone marrow MSCs [48], whereas urine‐derived induced MSCs show the opposite combination—strong immune privilege but weak suppressive function [49]. The practical lesson is that low immunogenicity does not guarantee therapeutic immunomodulation: the two properties must be assessed independently rather than collapsed into a single notion of immune compatibility.

**FIGURE 2:**
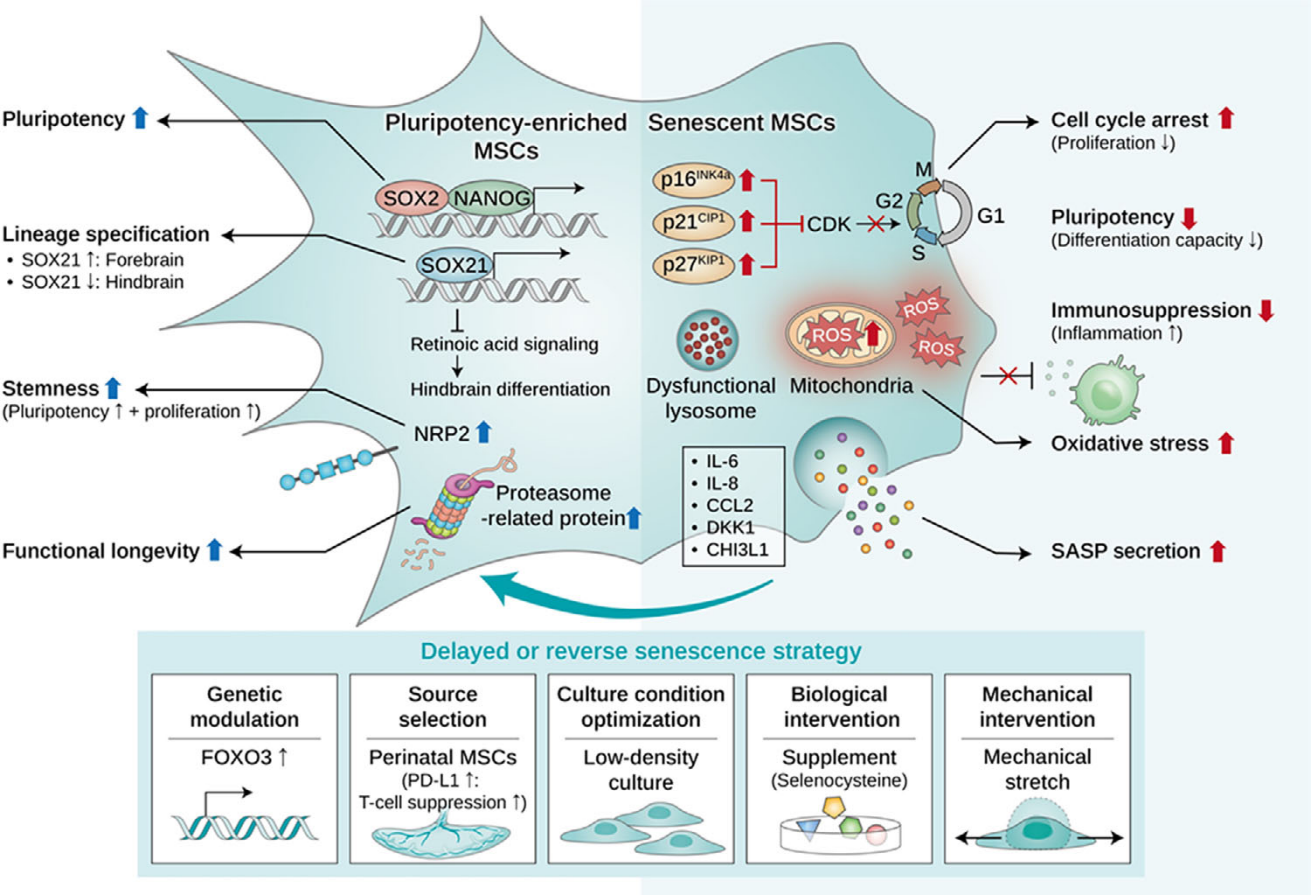
Pluripotency‐associated molecular signatures versus senescence programs define MSC functional fitness and immune‐modulatory potency. Conceptual schematic illustrating how pluripotency/stemness‐enriched MSC states (left) and senescent MSC states (right) differentially shape therapeutic performance. Pluripotency‐enriched MSCs are characterized by elevated expression of stemness‐associated regulators (e.g., SOX2, NANOG) and lineage‐bias factors (e.g., SOX21), with tissue/clone‐specific signatures that can influence differentiation trajectories (e.g., SOX21‐linked neuroectodermal patterning/retinoic acid–associated fate decisions). Enrichment of functional stemness markers such as NRP2 and proteostasis/proteasome‐related programs is associated with increased self‐renewal, proliferation, and functional longevity. In contrast, senescent MSCs exhibit upregulation of cell‐cycle inhibitors (p16^INK4a^, p21^CIP1^, p27^KIP1^) leading to CDK blockade and cell‐cycle arrest, accompanied by mitochondrial/lysosomal dysfunction, increased ROS‐driven oxidative stress, and a senescence‐associated secretory phenotype (SASP) rich in pro‐inflammatory and tissue‐degenerative mediators (e.g., IL‐6, IL‐8, CCL2, DKK1, CHI3L1). These senescence features collectively reduce pluripotency‐related capacity, impair differentiation and proliferation, and diminish immunosuppressive activity, thereby limiting therapeutic efficacy. The lower panel summarizes representative strategies to delay or reverse MSC senescence and preserve potency, including genetic modulation (e.g., FOXO3 activation), source selection (e.g., perinatal MSCs with higher PD‐L1 and stronger T‐cell suppression), culture optimization (e.g., low‐density expansion), biological interventions (e.g., antioxidant/metabolic supplementation), and mechanical conditioning (e.g., physiologic stretch). Original figure created by the authors. Copyright 2026, The Authors.

Given that the two axes are separable, the operational question is which variables shift each. Donor sex is one measurable determinant: female adipose MSCs exert markedly stronger IDO1‐dependent T‐cell suppression than male counterparts [50]. Inflammatory licensing is another, and a more controllable one—dual IFN‐γ/IL‐1β priming enhances suppressive and tissue‐reparative programs and improves outcomes in lung injury [51]. The most durable gains come from licensing that is heritably imprinted: in situ conditioning within a BMP‐2 osteo‐organoid installs DNA‐methylation changes at PGE2‐synthesis genes that confer an immunoregulatory “memory” persisting across passages [52], while umbilical cord lining MSCs illustrate the desired endpoint—combining low immunogenicity with robust, IFN‐γ‐responsive suppression [53]. The unifying point is practical rather than descriptive: because immunogenicity and immunomodulatory potency are engineerable endpoints rather than fixed traits, an allogeneic MSC product should be deliberately designed along both axes—through source choice, donor selection and defined priming—and characterized as such, rather than inferred from a single immunophenotyping panel (Figure 2).

#### 2.1.5 Metabolic Profiles and Mitochondrial Functions

The metabolic state and mitochondrial functionality of MSCs are pivotal determinants of their stemness, survival, and therapeutic efficacy. MSCs dynamically regulate energy production through a balance between glycolysis and oxidative phosphorylation (OXPHOS), accompanied by mitochondrial network remodeling, mitophagy, and reactive oxygen species (ROS) modulation. Beyond energy generation, mitochondrial dynamics and metabolic plasticity actively guide lineage commitment and functional performance. Mitochondrial fusion and biogenesis facilitate adipogenic and osteogenic differentiation, whereas fission and mitophagy predominate during chondrogenesis or stress adaptation.

Metabolic state is not a passive consequence of MSC phenotype but an active, upstream determinant of stemness, lineage choice and therapeutic potency. The organizing principle is that a defined bioenergetic configuration—oxidative phosphorylation–leaning metabolism with controlled reactive oxygen species and high mitochondrial quality—marks the fittest, longest–lived cells and is mechanistically required for their functions. Long–lived cord–blood MSC subpopulations are distinguished from short–lived ones precisely by higher mitochondrial DNA content, lower mitochondrial DNA methylation and OXPHOS reliance, and inhibiting mitochondrial complex activity is sufficient to drive them into senescence [54]; bioenergetic plasticity, the capacity to flexibly switch substrate use, further predicts which cord–derived MSCs survive ischemic stress [55]. That metabolism instructs rather than follows fate is demonstrated directly by mitochondrial dynamics: fusion and biogenesis enable the oxidative shift required for osteogenic and adipogenic commitment, whereas fission and mitophagy accompany chondrogenesis, and disrupting these transitions blocks the corresponding lineages [56].

**FIGURE 3:**
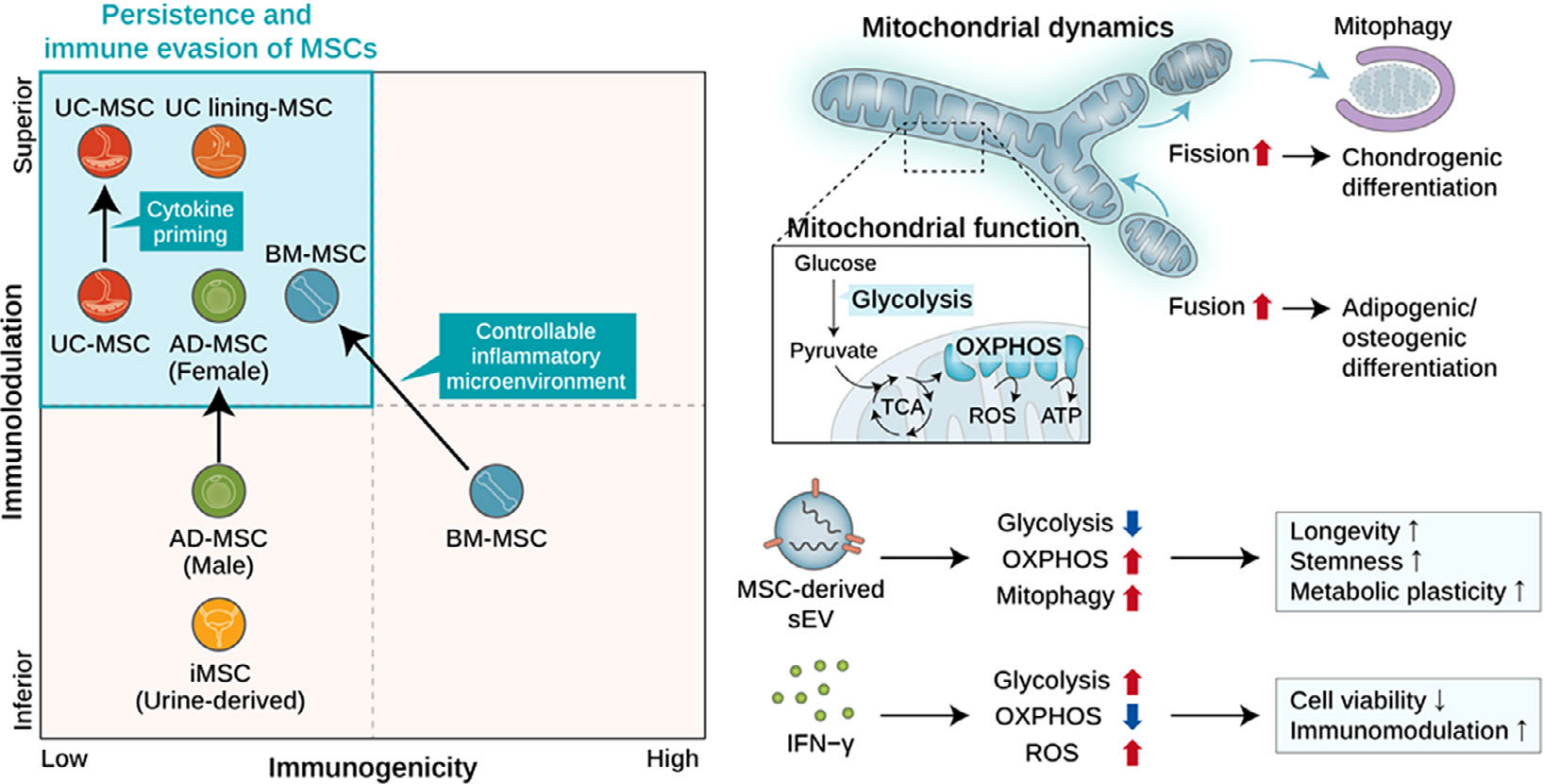
Metabolic–mitochondrial plasticity and immunobiology as convergent determinants of MSC functional fitness and therapeutic efficacy. Left panel illustrates the trade‐off between immunogenicity and immunomodulation across representative MSC platforms, emphasizing how tissue origin and donor factors (e.g., perinatal sources such as UC‐MSCs/UC lining‐MSCs, sex‐dependent differences in AD‐MSCs, and urine‐derived iMSCs) shape immune recognition, persistence, and immune‐evasive capacity. The schematic also highlights actionable approaches to enhance clinical performance, including cytokine priming to boost immunomodulatory activity and microenvironmental/in situ licensing within a controllable inflammatory niche to reinforce durable immunoregulatory function. Right panel summarizes how mitochondrial dynamics and bioenergetics actively regulate MSC fate and potency: mitochondrial fission and mitophagy are linked to stress adaptation and chondrogenic differentiation, whereas mitochondrial fusion/biogenesis supports elevated OXPHOS and promotes adipogenic/osteogenic differentiation. The lower diagrams provide example “metabolic intervention” axes relevant to therapeutic optimization: MSC‐derived small extracellular vesicles (sEVs) support mitochondrial quality control (e.g., mitophagy) and OXPHOS‐associated fitness features that correlate with longevity, stemness, and metabolic plasticity, while IFN‐γ licensing drives a shift toward aerobic glycolysis, increases mitochondrial ROS, and strengthens secretion of immunosuppressive mediators (e.g., IDO/PGE2), potentially at the expense of cell viability. Up/down arrows indicate the direction of relative changes in the depicted pathways and functional outputs. Original figure created by the authors. Copyright 2026, The Authors.

Because metabolism is both instructive and pharmacologically accessible, it constitutes a practical optimization target. Metabolic state is also tightly coupled to immunomodulation: IFN–γ licensing forces a shift to aerobic glycolysis that is itself required to sustain IDO and PGE2 secretion, so that suppressing glycolysis abolishes immunopotency [57]. These dependencies can be leveraged or restored therapeutically—small molecules such as quercetin modulate mitochondrial respiration and sirtuin expression in a dose– and age–dependent manner [9], and MSC–derived small extracellular vesicles can transfer mitochondrial quality–control machinery to repair dysfunctional metabolism in diabetic wound beds [58]. The same metabolic logic governs other adult stem cells, with quiescent, activated and aged muscle satellite cells occupying distinct metabolic states that gate regeneration [59]. The translational implication is that bioenergetic and mitochondrial fitness should be treated as an explicit, measurable, and tunable potency attribute rather than an incidental property of culture. These metabolic and mitochondrial determinants of MSC functional potency—together with their links to immunomodulatory activity—are summarized in Figure 3.

### 2.2 Microenvironmental Determinants

#### 2.2.1 Niche‐Derived Biochemical Signaling (Cytokines, Growth Factors, Chemokines)

The adult stem cell niche is a highly specialized microenvironment that governs stem cell behavior through a complex interplay of biochemical, biophysical, and cellular cues. Among these, soluble and matrix‐bound biochemical signals—such as cytokines, growth factors, and chemokines—play a central role in orchestrating stem cell fate decisions, including self‐renewal, quiescence, migration, lineage commitment, and differentiation. These signaling molecules are secreted or presented by diverse niche‐resident components, including stromal cells, endothelial cells, immune cells, and sensory neurons, and their effects are often modulated by spatial and temporal cues, as well as by synergistic interactions with mechanical and structural niche elements.

Soluble and matrix–bound biochemical cues—cytokines, growth factors, and chemokines—are classical instructors of stem cell fate, but the principle that best organizes recent niche studies is that their action is conditional: biochemical signals are interpreted through the geometric and mechanical context in which they are presented. The clearest demonstration is that substrate stiffness sets the boundaries within which biochemical ligands operate—soft matrices permit BMP2– and CCL2–driven commitment that stiff matrices suppress regardless of the ligand cocktail [60]—and that stem cell morphology itself gates signal reception, since intestinal stem cells must adopt a high surface–to–volume conical shape to receive Paneth–cell–derived Wnt and Notch cues efficiently [61]. Biochemical signaling is therefore not an autonomous input but one that is amplified or muted by physical niche features. The matrix architecture and mechanotransduction machinery through which this physical gating operates are examined in detail in Section 2.2.2.

**FIGURE 4:**
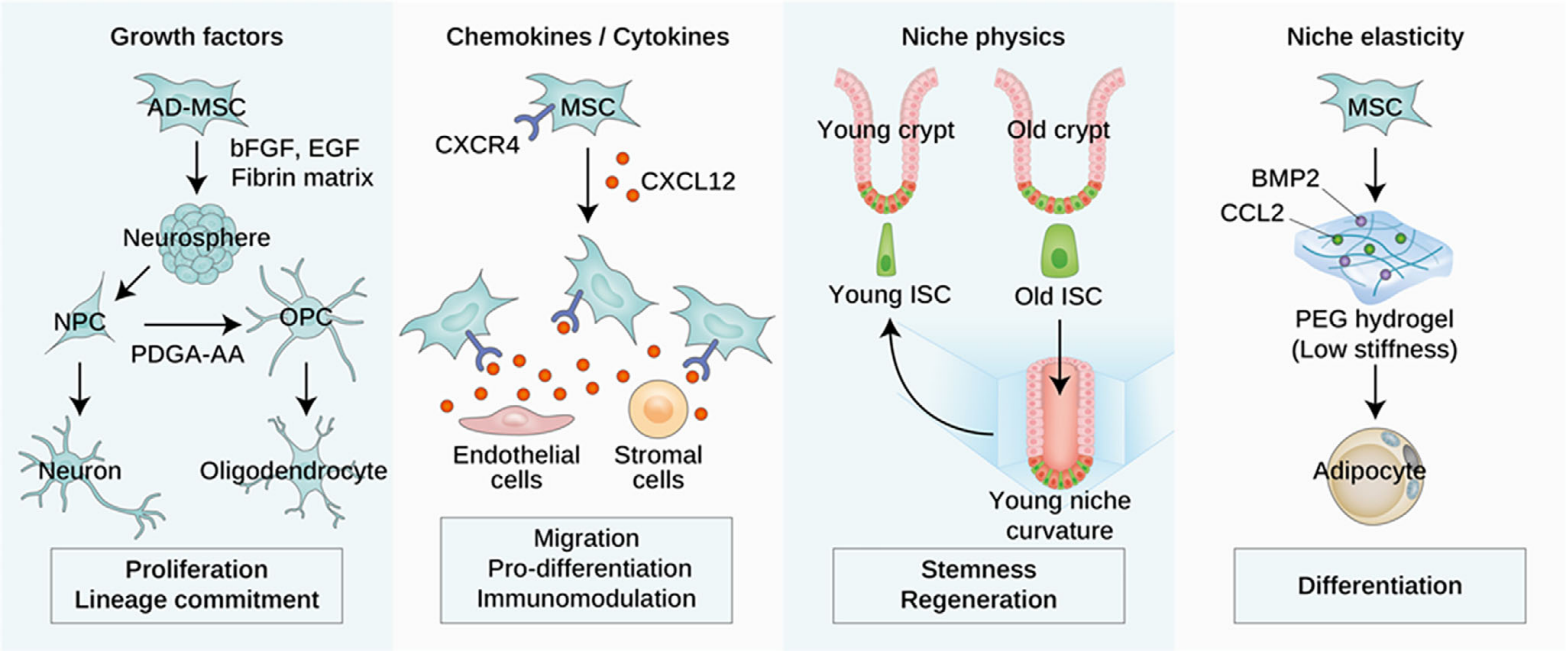
Niche‐derived biochemical cues and biophysical context cooperatively regulate adult stem cell fate and function. Schematic overview of how soluble and matrix‐presented signals (growth factors, cytokines, and chemokines) integrate with niche geometry and mechanics to control stem cell self‐renewal, migration, and lineage commitment. Left panel (Growth factors): A fibrin‐based biomimetic niche supplemented with EGF/bFGF supports progenitor expansion (neurosphere formation) and, with stage‐specific cues (e.g., PDGF‐AA), guides AD‐MSCs toward neural progenitor cells (NPCs) and oligodendrocyte progenitor cells (OPCs), enabling subsequent neuronal and oligodendroglial differentiation. Second panel (Chemokines/Cytokines): CXCL12 (SDF‐1) produced by endothelial and stromal niche cells signals through CXCR4 on MSCs to promote chemotaxis/migration, injury‐site recruitment, pro‐differentiation responses, and immunomodulatory effects. Third panel (Niche physics): Age‐ and structure‐dependent changes in tissue architecture (e.g., crypt/niche curvature) modulate stem cell morphology and the efficiency of receiving niche signals, influencing intestinal stem cell (ISC) stemness and regenerative capacity. Right panel (Niche elasticity): Substrate stiffness sets boundaries for responsiveness to biochemical ligands; in soft PEG hydrogels (low stiffness), factors such as BMP2 and CCL2 synergize to promote MSC lineage commitment (illustrated here as adipogenic differentiation). Boxes summarize dominant functional outputs per panel. Abbreviations: AD‐MSC, adipose‐derived mesenchymal stromal/stem cell; NPC, neural progenitor cell; OPC, oligodendrocyte progenitor cell; ISC, intestinal stem cell; PEG, poly(ethylene glycol). Original figure created by the authors. Copyright 2026, The Authors.

Within this integrated framework, individual niche factors still carry decisive, indication–relevant information. Sensory–nerve–derived FGF1 sustains the resident MSC pool through an FGFR1–mTOR–autophagy axis, linking tissue innervation to stem cell maintenance [62]; mesenchyme–derived neuregulin–1 drives intestinal stem cell proliferation more potently than EGF via ErbB2/3 signaling [63, 64]; matrix–bound EGF, bFGF and PDGF presented from a fibrin scaffold direct staged neural–lineage commitment through Wnt and Notch [64]; and the chemokine CXCL12/SDF–1, secreted by perivascular niche cells, governs MSC retention and injury–site recruitment [65]. The actionable synthesis for therapy design is that engineered niches must co–specify biochemical and physical cues: delivering a growth factor without matching matrix mechanics and construct geometry forfeits much of its instructive value. These niche–derived biochemical signals (cytokines, growth factors, and chemokines) and their integration with biophysical niche context are summarized in Figure 4.

#### 2.2.2 Extracellular Matrix Interactions and Biomechanical Cues

The extracellular matrix (ECM) constitutes a dynamic and instructive niche that regulates adult stem cell fate not only through its biochemical composition but also via its physical, structural, and mechanical properties. Beyond serving as a passive scaffold, the ECM integrates ligand presentation, architectural organization, and biomechanical forces to orchestrate cell adhesion, cytoskeletal organization, and nuclear signaling. Stem cells sense these cues through integrin‐mediated focal adhesions, mechanosensitive ion channels, and cytoskeletal tension, translating them into transcriptional programs that govern lineage specification, self‐renewal, and paracrine function.

The extracellular matrix is an instructive niche whose physical properties are transduced into transcriptional programs through a conserved integrin–cytoskeleton–nuclear axis, with YAP/TAZ acting as the principal mechanosensitive effectors. The principle that distinguishes recent work from a simple “stiffness determines fate” model is that multiple, partly independent physical features—rigidity, viscosity, topography, ligand conformation, fibril alignment and geometry—each feed this axis, and stiffness is not always the dominant input. Extracellular fluid viscosity redirects MSCs toward osteogenesis independently of substrate stiffness and leaves a persistent “mechanomemory” [66]; ECM fibril strain acts as a biomechanical switch that exposes cryptic integrin–binding sites and selects α5β1 versus αvβ3 usage [67]; and in trauma–induced heterotopic ossification, ECM alignment—more than stiffness—governs progenitor mechanotransduction and lineage choice [68].

The foundational principle that matrix mechanics is an instructive rather than permissive cue was established by the demonstration that substrate elasticity alone—independent of soluble factors—directs MSC commitment toward neurogenic, myogenic, or osteogenic fates [69], and that this specification becomes irreversible once cells dwell on a given stiffness long enough to accumulate a mechanical “memory” [70]. Subsequent work resolved its molecular basis: matrix stiffness is relayed to the nucleus, where lamin‐A levels scale with tissue elasticity and reinforce stiffness‐directed differentiation [71], while cell geometry and RhoA‐dependent cytoskeletal tension constitute a parallel, equally instructive input [72]. Critically, cells do not read bulk stiffness directly but sense how the matrix responds to force: substrate viscoelasticity and stress relaxation are now recognized as decisive parameters, with faster‐relaxing hydrogels markedly enhancing MSC spreading, proliferation, and osteogenesis [73, 74], and the tethering of adhesion ligands—rather than stiffness per se—governing fate on otherwise identical substrates [75]. In parallel, nanoscale surface topography offers an independent, chemistry‐free route to program fate: defined nanoscale disorder drives osteogenesis without inductive media [76], specific nanopatterns instead preserve MSC multipotency during expansion [77], and the integrin‐mediated basis of these responses now constitutes a coherent design framework [78].

A point that deserves greater emphasis is that the final step of mechanotransduction is the deformation of the nucleus itself, which behaves not as a passive endpoint but as an active mechanosensor. Cytoskeletal forces are transmitted across the nuclear envelope by the LINC complex, which physically couples the actin and intermediate‐filament networks to the nuclear lamina, so that matrix stiffness is converted into mechanical tension on the lamina and on chromatin. The lamin‐A scaling noted above is one expression of this coupling—the lamina stiffens on rigid substrates and thereby reinforces stiffness‐directed differentiation—but the same force transmission also deforms the nucleus directly, reorganizing chromatin, altering the accessibility of mechanoresponsive loci, and modulating the nuclear import of mechanosensitive transcriptional regulators such as YAP/TAZ and MRTF‐A. Viewed this way, the nucleus is the integrating node at which the biochemical cues of Section 2.2.1 and the matrix‐mechanical cues of the present subsection are jointly converted into a transcriptional decision. This convergence is also a translational opportunity: because nuclear‐envelope composition and lamina tension gate cell fate, they constitute at once a design target—substrate properties can be tuned to impose a defined nuclear‐mechanical state—and a candidate quality attribute, since lamina status reports where a cell lies between a primitive and a committed condition.

**FIGURE 5:**
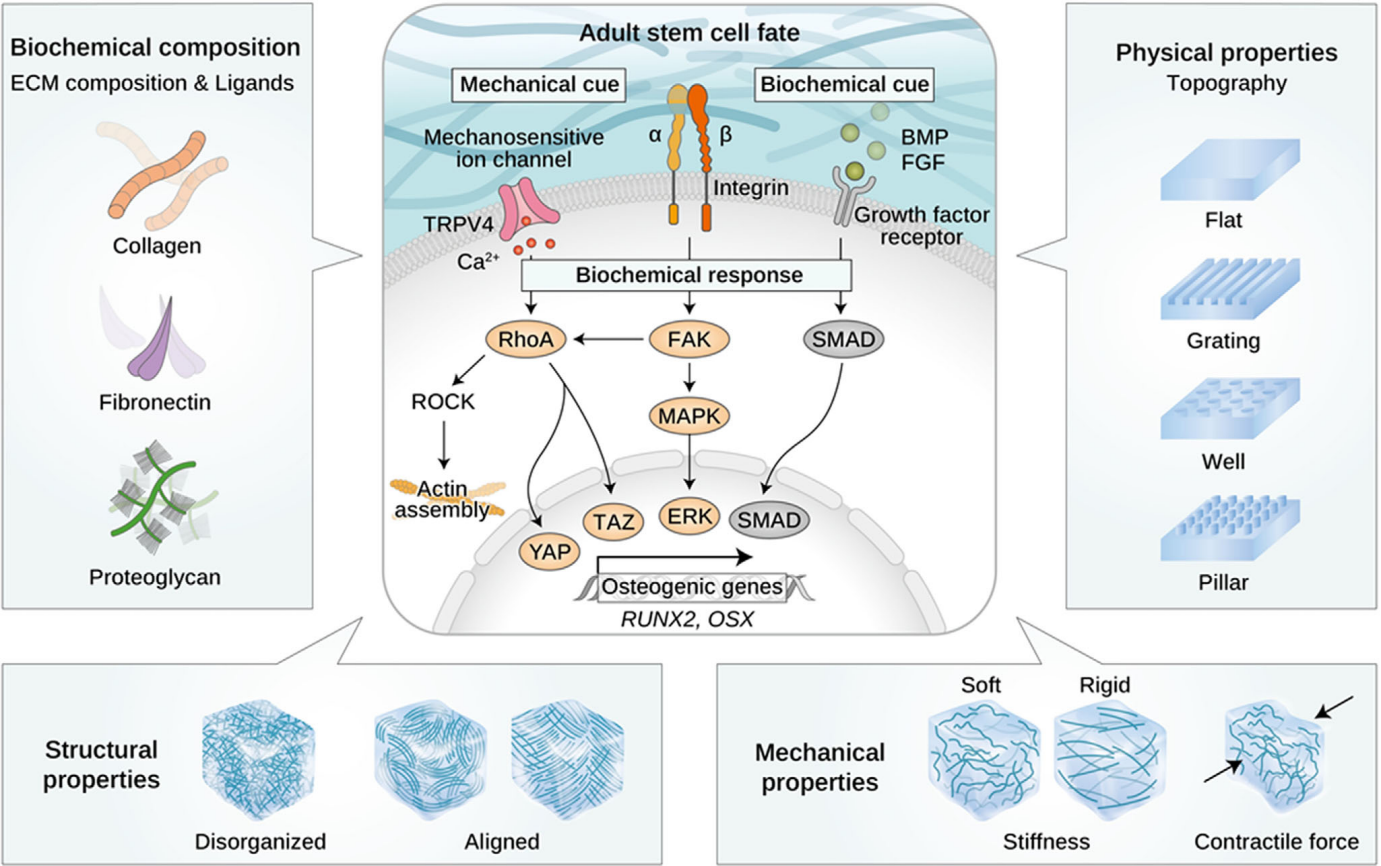
Extracellular matrix (ECM) interactions and biomechanical cues as instructive regulators of adult stem cell fate and function. Schematic summary illustrating how ECM composition, architecture, and mechanics are sensed through integrin‐based focal adhesions, cytoskeletal tension, and mechanosensitive pathways to control self‐renewal, lineage specification, and paracrine/immunomodulatory outputs. Representative examples include: (i) extracellular fluid viscosity as a stiffness‐independent cue that enhances actin remodeling and membrane tension, activates mechanosensitive Ca^2^^+^ entry (e.g., TRPV4) and RhoA/ROCK–YAP/RUNX2 signaling, and can induce persistent “mechanomemory” with concomitant immunomodulatory secretome changes; (ii) ECM ligand presentation combined with nanoscale topography (e.g., cell‐derived ECM on nanopatterns) that promotes focal adhesion maturation, stress fiber formation, and YAP nuclear translocation, potentiating osteogenic gene programs; (iii) combinatorial ECM assemblies across stiffness gradients that reprogram the MSC secretome and cytokine responses in a composition‐ and mechanics‐dependent manner; (iv) strain‐dependent fibronectin (Fn) remodeling, where cell‐generated tension exposes cryptic binding sites to bias integrin usage (α5β1 vs αvβ3) and growth factor receptor crosstalk (e.g., EGFR) to drive osteogenesis; (v) ligand identity × elasticity design rules in hematopoietic stem/progenitor cells (HSPCs), where early fate decisions are rapidly shaped by integrin engagement and actomyosin/ROCK‐dependent intracellular tension; (vi) ECM alignment/organization that gates FAK phosphorylation and YAP/TAZ activity to steer mesenchymal progenitor differentiation (e.g., toward osteogenesis vs adipogenesis) in injury contexts; and (vii) ECM microgeometry (e.g., microgrooves) that controls cell shape and focal adhesion patterning to activate FAK–MAPK/ERK signaling and enhance osteogenic maturation. Original figure created by the authors. Copyright 2026, The Authors.

Across these inputs the downstream logic converges: focal–adhesion maturation, actomyosin tension, and nuclear YAP/TAZ translocation integrate the physical signal and commit the cell, as shown for nanoscale topography combined with cell–derived ECM [79], microgroove geometry [80], and combinatorial ECM compositions that, gated by stiffness, reprogram the MSC secretome itself [81]. The same integrin–tension machinery operates rapidly in hematopoietic stem/progenitor cells, where ECM ligand identity and elasticity together dictate early fate within hours [82]. The translational value of this synthesis is a set of concrete biomaterial design rules: because nuclear mechanotransduction is the common convergence point, scaffolds can be engineered to preserve primitive progenitors or to drive a specific lineage by tuning whichever physical parameter—not necessarily bulk stiffness—most strongly engages that pathway in the target cell. These ECM–mediated interactions and biomechanical cues that instruct adult stem cell fate and function are summarized in Figure 5.

#### 2.2.3 Oxygen Tension and Hypoxic Conditioning Effects

Oxygen availability is a fundamental microenvironmental cue that shapes the biological behavior, metabolic state, and therapeutic efficacy of adult stem cells. In vivo, most stem cell niches are inherently hypoxic relative to atmospheric oxygen, with partial pressures tightly regulated to preserve stemness, modulate lineage commitment, and maintain long‐term regenerative capacity. In vitro, however, cells are often cultured under hyperoxic conditions (∼20% O_2_), which can disrupt this physiological balance, driving premature differentiation, altering metabolic programs, and impairing survival and functional integration after transplantation.

Most adult stem cell niches are physiologically hypoxic, so the conventional ∼20% O_2_ of standard culture is itself a non–physiological stressor; conditioning cells under controlled low oxygen is therefore best understood not as a perturbation but as a restoration of niche–relevant signaling. The unifying principle is that oxygen tension acts as a master regulator that simultaneously preserves stemness and reprograms the secretome and metabolism toward in vivo readiness, largely through HIF–1α. Hypoxia preserves progenitor immaturity while restraining premature osteogenic commitment yet sparing angiogenic competence [83], and acute hypoxic exposure rapidly stabilizes HIF–1α to upregulate SDF–1 and enhance directional homing—an immediately translatable engraftment strategy [84]. Hypoxic conditioning also sustains osteogenic competence epigenetically, by maintaining the cytosolic acetyl–CoA pool needed for histone acetylation at bone–gene loci [85], and reshapes the secretome toward pro–angiogenic, cytoprotective output [86].

**FIGURE 6:**
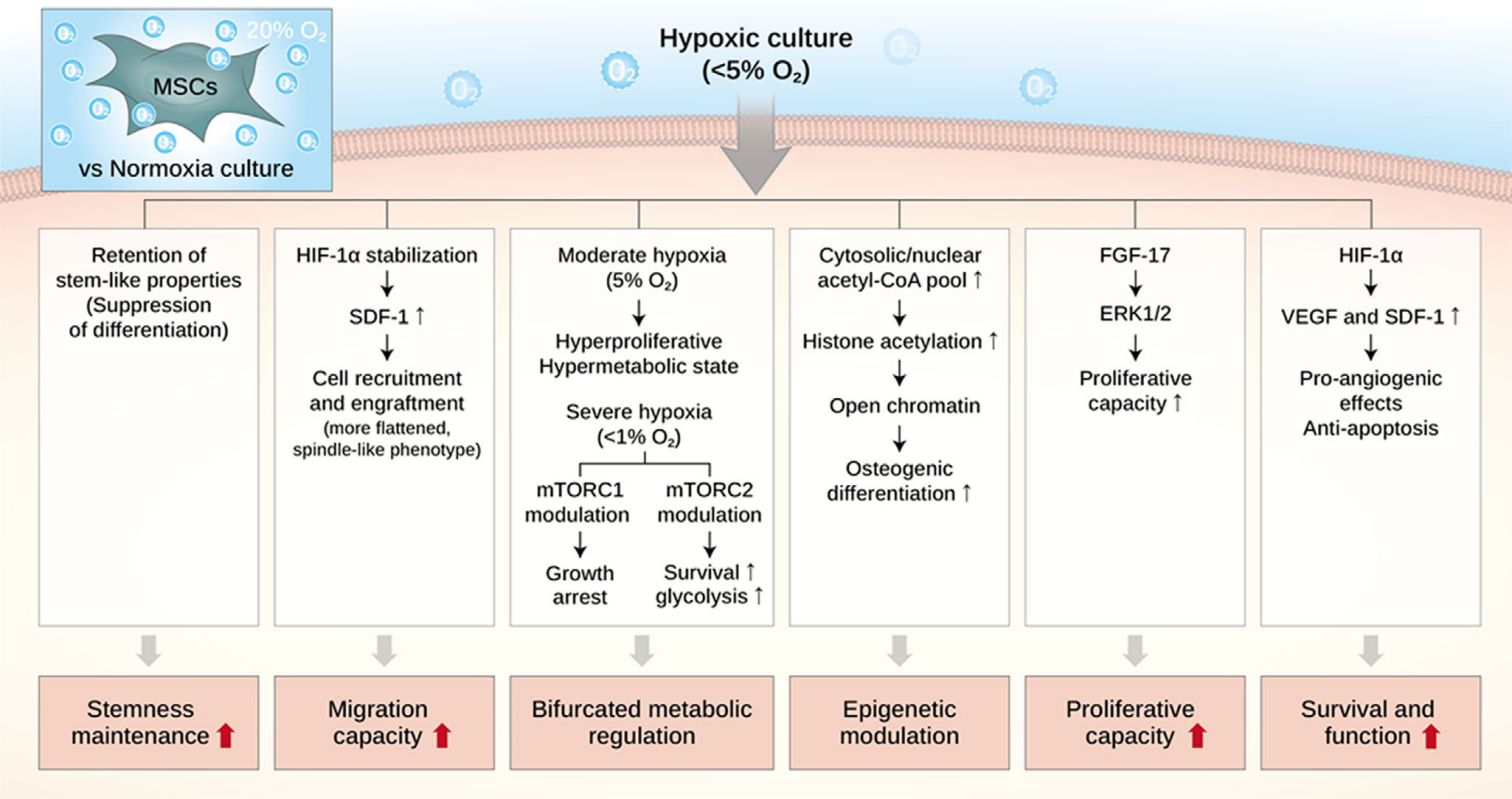
Oxygen tension and hypoxic conditioning as a master regulator of MSC potency. Schematic overview of how hypoxic culture (<5% O_2_), compared with conventional normoxia (∼20% O_2_), remodels mesenchymal stromal/stem cell (MSC) biology through coordinated effects on stemness, migration, metabolism, epigenetics, proliferation, and survival. Hypoxia can preserve stem‐like properties and suppress premature differentiation. HIF‐1α stabilization rapidly enhances SDF‐1/CXCL12 signaling, promoting a migratory, spindle‐like phenotype that improves cell recruitment and engraftment. Oxygen tension further drives bifurcated metabolic regulation: moderate hypoxia (∼5% O_2_) supports a hyperproliferative/hypermetabolic state, whereas severe hypoxia (<1% O_2_) favors a quiescence‐like survival program characterized by mTORC1 down‐modulation (growth restraint) and mTORC2‐associated survival with increased glycolysis. In parallel, hypoxia supports a metabolism–chromatin axis, increasing the cytosolic/nuclear acetyl‐CoA pool, enhancing histone acetylation, maintaining open chromatin, and thereby sustaining osteogenic competence. Hypoxic conditioning can also activate an autocrine FGF‐17–ERK1/2 loop to preserve proliferative capacity, and HIF‐1α–dependent induction of VEGF and SDF‐1 enhances pro‐angiogenic, anti‐apoptotic effects to improve overall cell survival and function. Boxes summarize the major functional outputs enhanced by hypoxic preconditioning. Original figure created by the authors. Copyright 2026, The Authors.

Two qualifications convert this principle into a usable protocol. First, the response is dose–dependent and non–monotonic: moderate hypoxia (∼5% O_2_) drives a hyperproliferative, biosynthetically active state whereas severe hypoxia (<1% O_2_) induces a quiescence–like survival program, with mTOR serving as the oxygen–sensing hub that partitions these outcomes [12]; expanding MSCs at moderate hypoxia from the point of isolation improves clonogenicity and biases lineage output without altering immunophenotype [87], and an autocrine FGF–17–ERK loop preserves proliferative capacity at late passage [88]. Second, chemical hypoxia mimetics such as CoCl_2_ do not faithfully reproduce gas–controlled hypoxia and can confound conditioning [12]. The translational synthesis is that hypoxic preconditioning is a powerful, low–cost potency lever, but only when oxygen concentration, exposure duration, and the choice of true versus mimetic hypoxia are specified as defined manufacturing parameters. These oxygen tension–dependent effects and hypoxic conditioning–driven changes in MSC stemness, migration, metabolism, and potency are summarized in Figure 6.

#### 2.2.4 Host Immune Cell Crosstalk and Inflammatory Microenvironment Modulation

The regenerative efficacy of adult mesenchymal stem cells (MSCs) is profoundly influenced by their dynamic interactions with host immune cells and the evolving inflammatory microenvironment. Beyond their intrinsic self‐renewal and multipotent differentiation capacities, MSCs function as active immunomodulators, engaging in reciprocal signaling with innate immune populations—most notably macrophages—to coordinate the sequential phases of tissue repair. This bidirectional crosstalk is highly context‐dependent, shaped by immune cell activation state, mode of interaction (paracrine versus direct contact), and the physicochemical properties of the surrounding extracellular matrix. Conversely, MSCs can reprogram immune cell phenotypes through soluble mediators, extracellular vesicles, and matrix remodeling signals, thereby steering the inflammatory niche toward a pro‐regenerative state.

The regenerative performance of an MSC graft is co–determined by its bidirectional dialogue with host immune cells—most prominently macrophages—so the inflammatory microenvironment should be treated not as a fixed backdrop but as a programmable variable that gates MSC fate and function. This crosstalk is genuinely reciprocal. On one side, the immune compartment instructs the MSC: M1–macrophage–derived TNF–α shapes MSC recruitment and immune phenotype [89], the cytokine balance of the niche licenses lineage choice—with macrophage IL–10 promoting osteogenesis [90]—and even dying macrophages broadcast instructions, delivering miR–155 via apoptotic vesicles that bias MSCs away from bone formation toward adipogenesis [91]. The mode of contact is itself a control parameter, since paracrine versus direct macrophage–MSC interaction tunes whether immunomodulatory or matrix–remodeling programs dominate [92].

**FIGURE 7:**
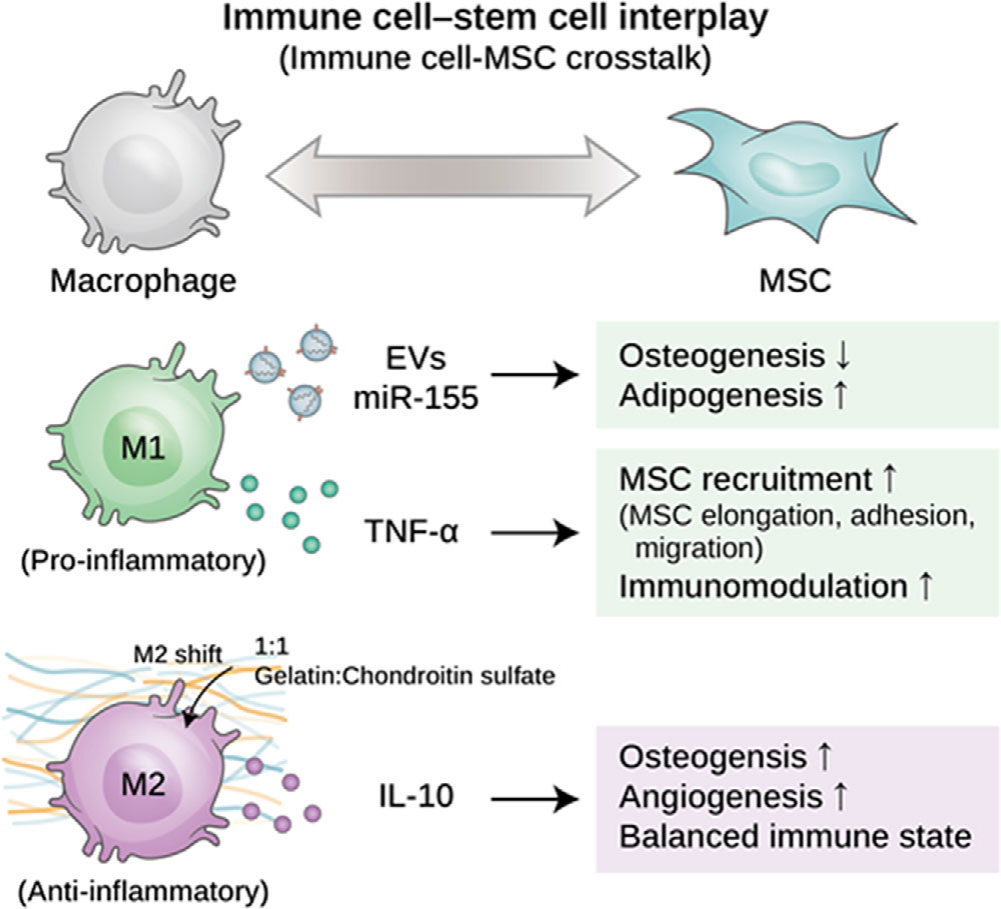
Immune cell–MSC crosstalk shapes the inflammatory niche and gates regenerative outcomes. Schematic illustrating the bidirectional interplay between macrophages and mesenchymal stromal/stem cells (MSCs) during tissue repair. In the early pro‐inflammatory phase, M1 macrophages provide soluble cues such as TNF‐α that can enhance MSC recruitment and migratory readiness (elongation, adhesion, migration) while modulating MSC immunoregulatory programs. In parallel, macrophage‐derived extracellular vesicles (EVs)/apoptotic vesicles carrying inflammatory microRNAs (e.g., miR‐155) can reprogram MSC fate, biasing differentiation toward reduced osteogenesis and increased adipogenesis. During the resolution/pro‐regenerative phase, M2 macrophages secrete anti‐inflammatory mediators such as IL‐10, which support a balanced immune state and promote osteogenesis and angiogenesis. Original figure created by the authors. Copyright 2026, The Authors.

On the other side, MSCs actively remodel the inflammatory niche toward resolution: they reprogram macrophage exosomal cargo to dampen M1 inflammatory programs [93], and MSC–secreted factors such as HGF shift the cytokine milieu from pro– to anti–inflammatory while redirecting endogenous repair, as shown after spinal cord injury [94]. Because this dialogue is bidirectional and tunable, it can be engineered from either direction—by priming MSCs, by reinforcing the secreted–factor axis through HGF overexpression to amplify tissue repair [95], or by designing biomaterials whose ECM composition steers host macrophages toward a pro–regenerative M2 state and thereby creates an instructive niche even without exogenous cells [96]. The translational synthesis is that controlling the inflammatory microenvironment—its phase, its dominant cytokines and the macrophage polarization state—is as decisive a lever for MSC efficacy as modifying the cells themselves. These host immune cell–MSC interactions and inflammatory microenvironment–dependent effects on regenerative outcomes are summarized in Figure 7.

## 3 Integrated Approaches to Improve Therapeutic Efficacy of Adult Stem Cells

**FIGURE 8:**
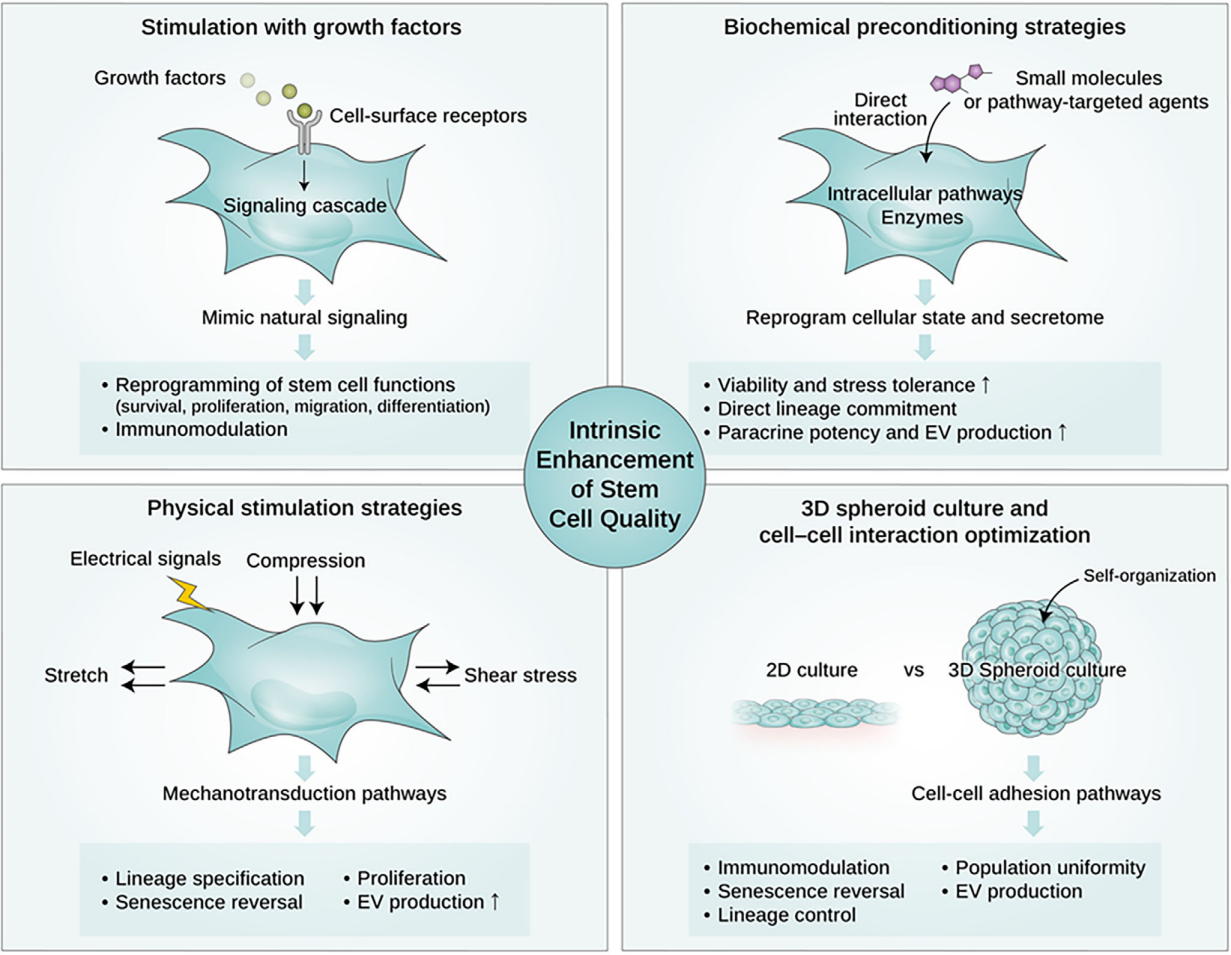
Bioengineering strategies to intrinsically enhance adult stem cell quality and therapeutic potency. Conceptual overview of four complementary bioengineering modalities that “pre‐program” adult stem cells (e.g., MSCs/NSCs) toward improved functional fitness prior to or after delivery. Top left: Growth factor stimulation activates cell‐surface receptors to mimic native niche signaling, thereby reprogramming survival, proliferation, migration, and differentiation programs while strengthening immunomodulatory activity. Top right: Biochemical preconditioning uses small molecules or pathway‐targeted agents that act directly on intracellular enzymes/signaling networks to transiently reset cellular state and secretome, increasing stress tolerance, directing lineage commitment, and enhancing paracrine potency including extracellular vesicle (EV) output. Bottom left: Physical stimulation (electrical signals, stretch, compression, and shear stress) engages mechanotransduction pathways to drive lineage specification, boost proliferation, promote senescence reversal, and increase EV production. Bottom right: 3D spheroid culture and cell–cell interaction optimization leverages self‐organization and adhesion‐mediated signaling to improve population uniformity, reinforce immunomodulation, enable senescence reversal and lineage control, and augment EV production. The central node highlights that these approaches converge on intrinsic enhancement of stem cell quality to yield more reproducible regenerative performance. Original figure created by the authors. Copyright 2026, The Authors.

Equally decisive is the context into which cells are placed. Spatially organized biofabrication and microenvironmental engineering convert adult stem cell therapy from a suspension infusion into an architected, instructive niche. Advances in 3D bioprinting show that precise control of geometry, stiffness, and factor presentation can direct cell fate and function—stabilizing survival, aligning angiogenesis with tissue architecture, and enabling rapid functional integration. Lattice, banded, tubular, and lamellar prints deliver heterotypic interfaces and diffusion‐aware layouts that program vascularization, epithelial–stromal organization, and even directed neural circuit formation, illustrating how “place” and “neighbors” are themselves therapeutics [18, 26, 27, 28, 29]. These orthogonal design knobs—material, mechanics, geometry, and spatially resolved cargo—provide a scalable route to standardize the host context that adult stem cells will encounter (Figure 8).

### 3.1 Intrinsic Enhancement of Stem Cell Quality

#### 3.1.1 Stimulation With Growth Factors

Exogenous growth factors and cytokines provide a powerful, programmable layer of control over adult stem cells, enabling precise recalibration of their therapeutic phenotype prior to or after transplantation. By engaging cognate receptors and rewiring downstream RTK–MAPK/ERK, PI3K–AKT, JAK–STAT, and SMAD pathways, these cues enhance cell survival, proliferation, motility, and homing; gate lineage commitment and matrix remodeling; and, critically, amplify paracrine immunomodulation and pro‐regenerative secretomes (including extracellular vesicles). Such signaling does not act in isolation: ligand‐induced cascades also reshape the inflammatory set‐point of host tissues, attenuating cytotoxic cytokines while promoting anti‐inflammatory mediators, angiogenesis, and tissue‐specific repair. In the following subsections, we synthesize evidence—spanning direct ligand priming, growth‐factor‐mediated licensing, and targeted augmentation of growth‐factor axes—demonstrating how tuned stimulation can simultaneously reprogram MSC‐intrinsic functions and the host inflammatory niche, thereby yielding reproducible gains in in vivo repair efficacy.

Exogenous growth factors constitute a programmable and reversible control layer over adult stem cells: by engaging cognate receptors and rewiring RTK–MAPK/ERK, PI3K–AKT, JAK–STAT, and SMAD signaling, they recalibrate survival, proliferation, homing, lineage commitment, and paracrine output before or after transplantation. The principle that should organize this literature, however, is context–specificity: the therapeutic effect of a given factor is not generic but is determined by the recipient cell type, the factor combination, and the timing of exposure. The same FGF family illustrates this clearly—FGF2 drives ligament/tendon specification of amniotic MSCs under a miR–16a–5p post–transcriptional brake [97], whereas a brief FGF–1/FGF–2 regimen commits adipose MSCs toward a preadipocyte fate while preserving stemness, and substituting EGF reverses this bias toward osteo– and chondrogenesis [98]. Growth–factor stimulation is therefore best understood as a tunable dial rather than a uniform potency boost.

Within this framework, individual factors deliver reproducible, mechanism–anchored gains. IGF–1 supports osteogenic commitment in gingiva–derived MSC spheroids [99] and in bone marrow MSCs through a MAPK/ERK–SOX4 axis [100], while MSC–derived EGF acts as a paracrine effector that simultaneously accelerates epithelial repair and dampens pathogenic cytokine and mast–cell responses in atopic dermatitis [101]. Critically, growth–factor cues reprogram not only the stem cell but also the host inflammatory set–point: EGF/bFGF priming of neural stem cells improves post–transplant survival, migration, and neuronal commitment [102], and melatonin priming engages an autocrine TGF–β feed–forward loop that enhances proliferation and metabolic–disease efficacy [103]. The translational synthesis is that growth–factor stimulation should be specified as a defined preconditioning protocol—factor identity, dose, combination, and duration—because these parameters, not the mere presence of a ligand, determine whether the intervention reprograms cells productively.

#### 3.1.2 Biochemical Preconditioning Strategies

Biochemical preconditioning refers to transient, ex vivo exposure of adult mesenchymal stem cells (MSCs) to small molecules or pathway‐targeted agents that reprogram cellular state and the secretome before delivery. Across diverse tissue contexts, short, well‐titrated regimens can (i) reinforce viability and stress tolerance, (ii) bias lineage programs in a context‐appropriate manner, and (iii) amplify paracrine potency—including extracellular vesicle (EV) yield and cargo quality—without eroding MSC identity. Given manufacturing demands, protocols that are simple (single‐agent), compatible with serum‐limited conditions, and yield durable, washout‐resistant benefits are preferable.

Biochemical preconditioning—transient ex vivo exposure to small molecules or pathway–targeted agents—reprograms cell state and the secretome before delivery. The unifying principle across otherwise unrelated agents is that brief, well–titrated regimens can durably reinforce stress tolerance, redirect lineage and amplify paracrine potency without eroding MSC identity, and that protocols which are simple, single–agent and washout–resistant are therefore preferable for manufacture. A recurring and translationally important theme is that several of these agents converge on the same downstream output—enhanced extracellular–vesicle (EV) production and cargo quality. Metformin amplifies EV yield and confers osteogenic, anti–inflammatory potency that persists under inflammatory stress [104], and a Wnt/β–catenin agonist (CHIR99021) increases exosome output through a SNAP25–dependent exocytic shift while enriching pro–regenerative cargo, indicating that preconditioning is also a route to cell–free therapeutics.

**FIGURE 9:**
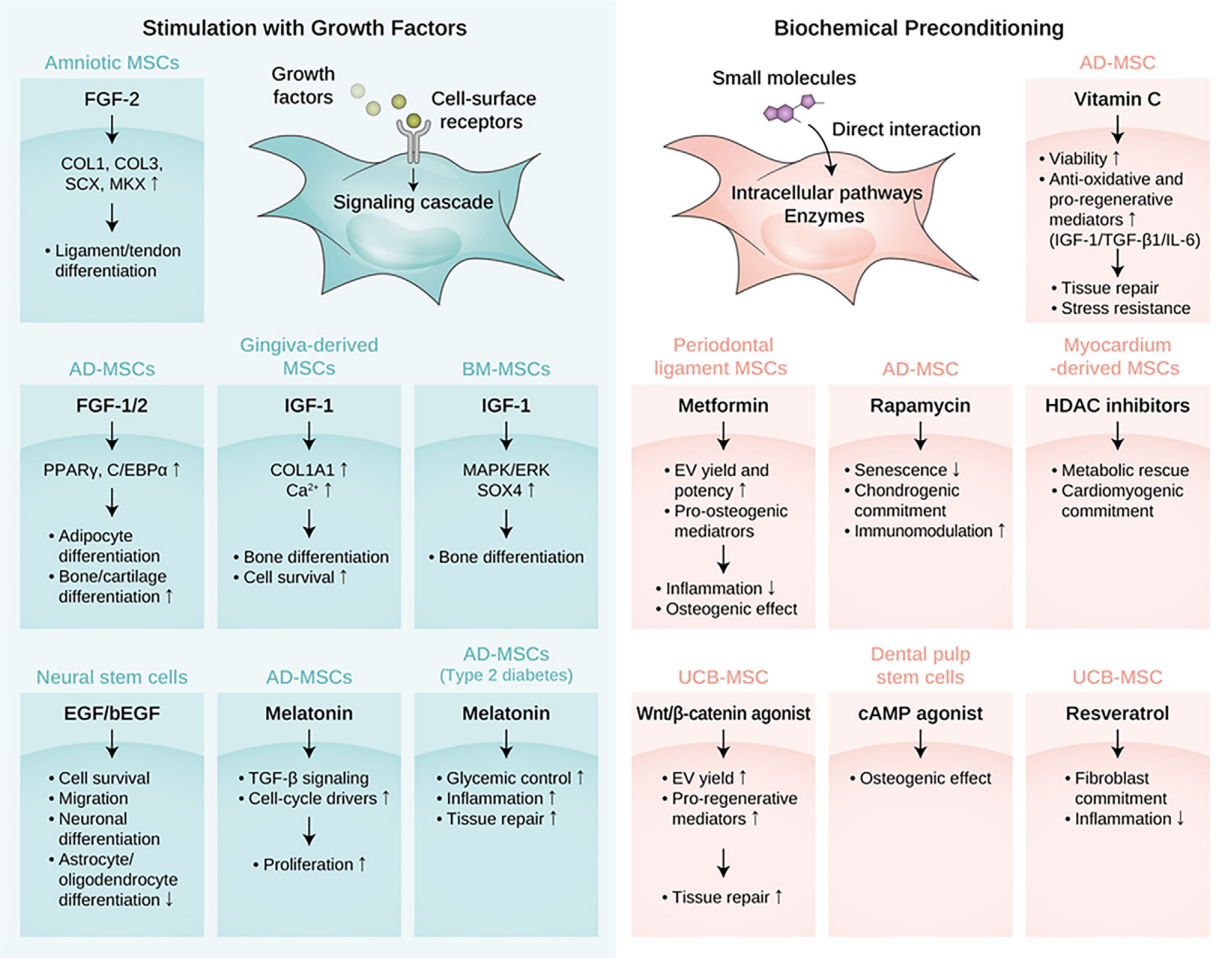
Intrinsic enhancement of stem cell quality through growth‐factor stimulation and biochemical preconditioning. Schematic summary of representative strategies that reprogram adult stem cells ex vivo to improve survival, lineage control, and paracrine potency prior to therapeutic application. Left panel (Stimulation with Growth Factors): Exogenous growth factors engage cell‐surface receptors to activate downstream signaling cascades (e.g., RTK–MAPK/ERK, PI3K–AKT, SMAD/JAK–STAT), thereby tuning fate and function in a context‐dependent manner. Examples include FGF‐2–driven ligament/tendon specification of amniotic MSCs (↑COL1, ↑COL3, ↑SCX, ↑MKX), FGF‐1/2 priming of AD‐MSCs toward adipogenic commitment (↑PPARγ, ↑C/EBPα) with reduced osteo/chondrogenic tendency, and IGF‐1–mediated osteogenic support in gingiva‐derived MSC spheroids (↑COL1A1, ↑Ca^2^^+^ deposition; improved viability) and BM‐MSCs (MAPK/ERK→↑SOX4; enhanced osteogenesis). EGF/bFGF stimulation promotes neural stem cell survival, migration, and neuronal differentiation while reducing astrocytic/oligodendrocytic differentiation. Melatonin priming enhances AD‐MSC proliferation via TGF‐β–associated cell‐cycle programs and improves therapeutic performance in metabolic disease settings by reducing inflammation and supporting tissue repair. Right panel (Biochemical Preconditioning): Short‐term exposure to small molecules directly modulates intracellular enzymes and pathways to reinforce stress tolerance, rejuvenate senescence‐associated decline, and enhance secretome/EV potency. Examples include vitamin C priming of AD‐MSCs (↑viability; ↑IGF‐1/TGF‐β1/IL‐6; improved stress resistance), metformin priming of periodontal ligament MSCs (↑EV yield/potency; ↓inflammation; enhanced osteogenic effects), rapamycin conditioning (↓senescence; ↑chondrogenic commitment; ↑immunomodulation), HDAC inhibitor priming of myocardium‐derived MSCs (metabolic rescue; cardiomyogenic commitment), Wnt/β‐catenin agonist conditioning of UCB‐MSCs (↑EV output; enriched pro‐regenerative mediators; improved tissue repair), cAMP agonist priming of dental pulp stem cells (enhanced osteogenesis), and resveratrol conditioning of UCB‐MSCs (fibroblast‐like/matrix‐productive bias; ↓inflammatory signaling). Arrows denote directionality of phenotypic or functional change. Original figure created by the authors. Copyright 2026, The Authors.

Beyond EV amplification, small–molecule priming tunes cell state along defined axes. Antioxidant priming with L–ascorbic acid selectively enriches reparative growth factors in the secretome while preserving viability [105]; rapamycin attenuates senescence and fibrosis and amplifies inflammatory licensing [106]; histone–deacetylase inhibitors rescue bioenergetic deficits and drive cardiomyogenic commitment, most strongly in disease–derived MSCs [107]; forskolin–mediated cAMP elevation enhances osteogenesis [108]; and resveratrol biases cells toward a fibroblastic, matrix–productive phenotype via PI3K/AKT [109]. Because each agent carries a defined toxicity threshold, the translational requirement is precision dosing: biochemical preconditioning is a powerful potency lever, but only when concentration, exposure window and cell state are specified as controlled manufacturing parameters. These representative approaches for intrinsically enhancing adult stem cell quality—through growth–factor stimulation and biochemical preconditioning—are summarized in Figure 9.

#### 3.1.3 Physical Stimulation Strategies

Physical stimulation leverages mechanical and electrical cues to reprogram adult stem cells without adding drugs or genetic modifiers, offering a scalable route to enhance potency while preserving identity. Across the studies summarized below, precisely dosed inputs—ranging from mechano‐electrical signaling on piezoelectric nanofibers, microfluidic deformation (“cell squeezing”), low‐magnitude shear stress, and physiologic cyclic stretch to electrical stimulation on soft, conductive matrices and mechanically defined microenvironments (stiffness/geometry)—consistently imprint durable pro‐regenerative states.

Physical stimulation reprograms adult stem cells through mechanical and electrical cues alone, offering a drug–free, additive–free and readily scalable route to enhanced potency. The principle that unifies these approaches is that precisely dosed physical inputs are decoded by mechanotransduction and electro–signaling into durable transcriptional states—a persistent “mechanical memory” that outlasts the stimulus. Crucially, distinct physical modalities engage distinct pathways and therefore steer fate predictably: on piezoelectric nanofiber scaffolds, electrical stimulation drives neuronal differentiation through a TRPC1–Akt–Wnt/β–catenin cascade whereas mechanical stimulation promotes glial fates through TRPV4–RhoA/ROCK, with minimal crosstalk between the two [110]. Physical conditioning is thus a programmable input whose outcome is set by the modality and its dosing. At the device level, nanoscale mechanical stimulation can be delivered at scale: a nanovibrational bioreactor applying nanometre‐amplitude, high‐frequency displacements drives 3D osteogenesis of MSCs without osteoinductive media or topographical patterning [111].

Across modalities, physical cues reproducibly enhance the two outputs most relevant to therapy—cell fitness and paracrine potency. Cyclic mechanical stretch reactivates antioxidant and DNA–repair programs to rejuvenate MSCs from aged donors [112], and paracrine priming likewise reverses aging hallmarks and extends survival in vivo [113], while low–magnitude shear stress “purinome–primes” osteogenic commitment via P2×7/P2Y6 signaling [114]. In parallel, physical inputs are an efficient means of upscaling EV–based products: microfluidic “cell squeezing” increases small–EV secretion roughly fourfold without compromising viability [115]. Mechanically and electrically defined microenvironments extend the same logic to lineage maturation, with soft conductive matrices imprinting a pro–neurogenic phenotype [116] and geometry–plus–stiffness confinement maturing stem–cell–derived cardiomyocytes [117]. The translational value of physical stimulation is therefore its combination of GMP–compatible simplicity with durable, mechanism–defined reprogramming—provided the stimulus waveform, magnitude, and duration are specified as controlled parameters.

#### 3.1.4 3D Spheroid Culture and Cell–Cell Interaction Optimization

3D spheroid culture exploits direct cell–cell adhesion and self‐produced matrix cues to reprogram adult stem cells toward a more potent, uniform, and clinically resilient state than is achievable in planar culture. Brief scaffold‐free aggregation synchronizes heterogeneous, passage‐expanded populations into compact clusters that amplify immunoregulatory and trophic secretomes, enhance homing and engraftment behavior, and improve safety profiles after systemic delivery. Optimizing cell–cell interaction geometry further elevates performance. In microphysiological 3D co‐cultures, pericyte‐like MSC subsets self‐organize along curvature‐defined vascular niches to smooth parent vessels and drive sprout elongation via rapid endothelial–mesenchymal ligand–receptor signaling. Procedurally, preserving multicellular clusters during passaging (rather than single‐cell dissociation) maintains gap‐junctional communications and various signaling pathways, supporting survival and neurogenic programming.

Scaffold–free 3D spheroid culture exploits direct cell–cell adhesion and self–produced matrix cues to convert heterogeneous, planar–expanded MSCs into a more potent, uniform, and clinically resilient state. The organizing principle is that restoring 3D cell–cell communication simultaneously achieves three goals that planar culture cannot: it standardizes a heterogeneous population, it amplifies the reparative secretome, and it reverses culture–acquired senescence. Brief spheroid aggregation collapses multiple planar subpopulations into a small number of immunosuppressive, trophic–factor–enriched clusters, improving T–cell suppression, pulmonary first–pass transit and homing after systemic delivery [118], and a short 3D–then–replating cycle reverses replicative–senescence hallmarks through mTOR relocalization and TFEB–driven autophagy without eliminating senescent cells [119].

**FIGURE 10:**
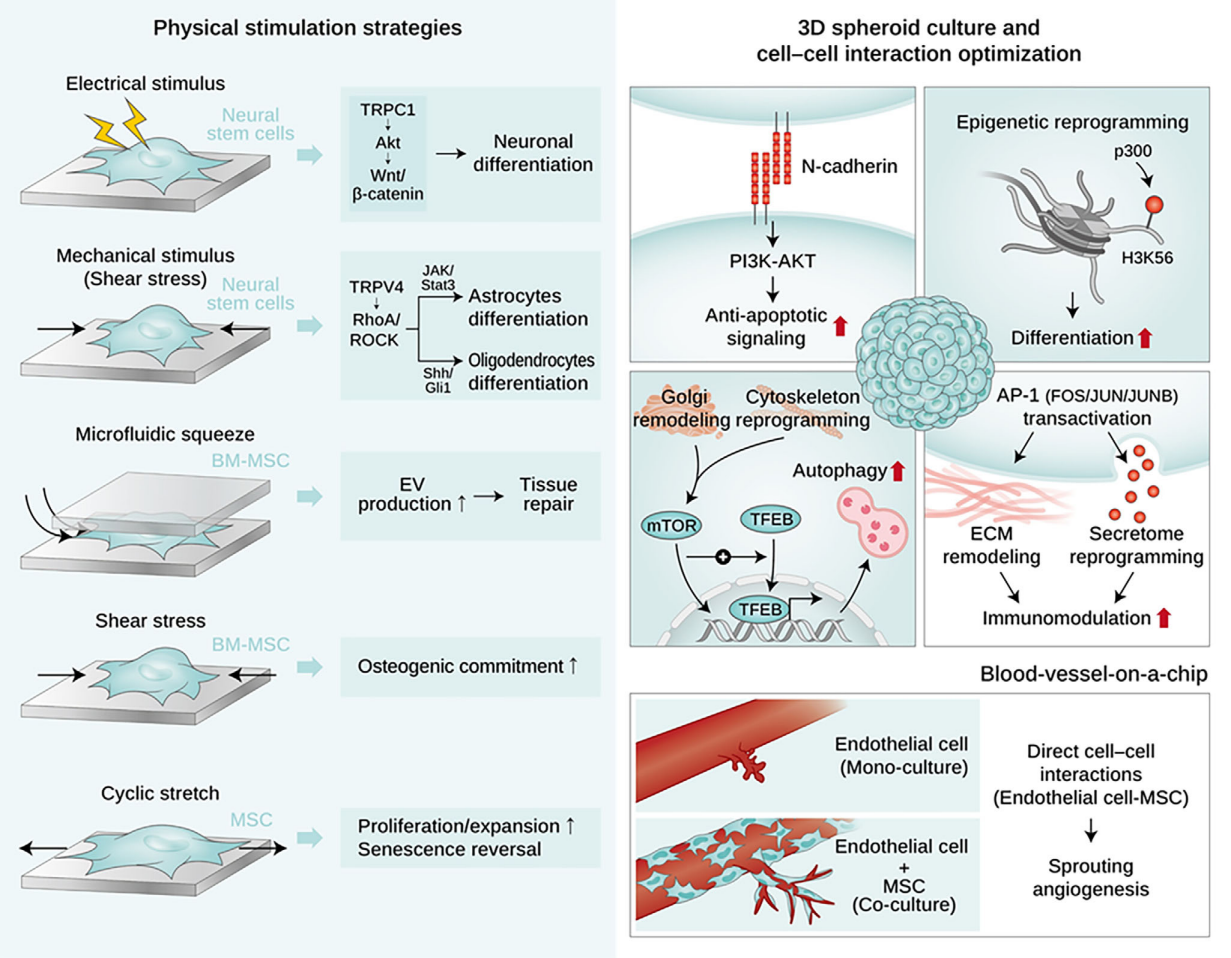
Bioengineering strategies to enhance adult stem‐cell potency through physical stimulation and 3D cell–cell interaction optimization. Physical stimulation strategies reprogram stem‐cell fate and functional outputs via mechanotransduction and electrosignaling. Electrical stimulation promotes neurogenic differentiation of neural stem cells through the TRPC1–Akt–Wnt/β‐catenin axis, whereas mechanical stimulation (e.g., shear stress) biases glial commitment via TRPV4–RhoA/ROCK signaling with downstream activation of JAK/STAT3 (astrocytic differentiation) and Shh/Gli1 (oligodendrocytic differentiation). Microfluidic “cell squeezing” increases extracellular vesicle (EV) production to augment tissue repair. Shear‐stress conditioning enhances osteogenic commitment, and cyclic stretch improves proliferation/expansion while supporting senescence reversal, collectively imprinting durable pro‐regenerative cellular states. 3D spheroid culture and cell–cell interaction optimization strengthen survival and therapeutic functions by reinforcing cell–cell adhesion and activating pro‐survival/quality‐control and transcriptional programs. N‐cadherin–mediated contacts activate PI3K–AKT anti‐apoptotic signaling. Spheroid formation induces Golgi and cytoskeletal reprogramming and engages mTOR–TFEB–autophagy pathways, improving stress resistance and functional maintenance. In parallel, 3D organization supports epigenetic reprogramming (e.g., p300‐dependent H3K56 acetylation) that facilitates differentiation capacity, and drives AP‐1 (FOS/JUN/JUNB)‐linked ECM remodeling and secretome reprogramming, enhancing immunomodulatory potency. A blood‐vessel‐on‐a‐chip model illustrates that direct endothelial cell–MSC interactions in 3D co‐culture promote sprouting angiogenesis compared with endothelial monoculture, highlighting how engineered microphysiological environments leverage optimized cell–cell coupling to improve regenerative outcomes. Original figure created by the authors. Copyright 2026, The Authors.

Mechanistically, the benefits of 3D organization are traceable to defined adhesion– and chromatin–level programs. N–cadherin–mediated contacts activate AKT–centered anti–apoptotic signaling that preserves viability even through ice–free supercooling preservation [120], and 3D culture engages p300–dependent H3K56 acetylation to augment hepatogenic differentiation [121], demonstrating that spatial context reaches the epigenome. Optimizing cell–cell interaction geometry further elevates performance: in microphysiological co–cultures, pericyte–like MSCs occupy curvature–defined vascular niches to smooth parent vessels and drive sprout elongation [122], and preserving multicellular clusters—rather than single–cell dissociation—during passaging maintains gap–junctional signaling and MAPK–dependent neurogenic programming in neural progenitors [123]. The translational significance is direct: because donor–to–donor and passage–related heterogeneity is a principal cause of inconsistent clinical outcomes, spheroid culture functions as a practical standardization and potency–enhancement step. The major bioengineering strategies that enhance adult stem–cell function—including physical stimulation–based approaches and 3D spheroid/cell–cell interaction optimization, along with their key mechanistic pathways and functional outcomes—are summarized in Figure 10.

### 3.2 Strategies to Enhance Stem Cell Functions With Bioengineering Technology

#### 3.2.1 Injectable Hydrogels and Biomimetic Scaffolds

Injectable hydrogels and biomimetic scaffolds provide a deployable, niche‐design toolkit that protects transplanted stem cells, programs their fate, and actively reshapes the host microenvironment. Macroporous matrices with spatially localized rigidity decouple pore‐wall stiffness from bulk softness to deliver persistent mechanotransductive cues (F‐actin organization, nuclear YAP) while preserving mass transport and injectability; conversely, time‐programmed mechanics—early soft, later stiff—leverages the mechanical memory of stem/progenitor cells to maintain proliferative competence and avert premature differentiation. Operationally, high‐performing systems share practical features essential for translation: shear‐thinning/self‐healing injectability, macroporosity for rapid cell–matrix integration, biodegradation synchronized to new tissue deposition, and chemically defined compositions that support both stromal and endothelial organization.

Injectable hydrogels and biomimetic scaffolds provide a deployable niche–design toolkit that protects transplanted cells, programs their fate through material properties, and actively reshapes the host microenvironment. The design principle that distinguishes high–performing systems is decoupling—the deliberate separation of material parameters so that each can be tuned independently of the others. Spatial decoupling is exemplified by shell–hardened and nanoparticle–reinforced macroporous hydrogels, which raise pore–wall stiffness to deliver persistent osteoinductive mechanotransduction while keeping the bulk matrix soft and mass–transport–permissive [124, 125]. Temporal decoupling is exemplified by time–programmed mechanics: because skeletal–muscle stem cells acquire a RhoA–dependent “mechanical memory” within a narrow early window, scaffolds that present soft cues first and stiffen later preserve progenitor competence and avert premature differentiation [126].

These design principles rest on a foundational body of biomaterials research establishing the hydrogel as an active instructor of transplanted‐cell fate. Formulations that tune a cell's capacity to mechanically remodel and exert traction on its matrix were shown to control MSC commitment [127], and—critically for translation—injectable, void‐forming hydrogels that decouple pore formation from stiffness governed the bone‐forming activity of transplanted MSCs in vivo, bridging in vitro mechanobiology to therapeutic outcome [128]. The temporal dimension is equally consequential: dynamically stiffening hydrogels revealed that cells integrate mechanical signals over time [129], and in 3D covalently crosslinked networks it is matrix degradability—by permitting cell‐generated traction—rather than initial stiffness that dictates MSC fate [130].

Beyond mechanics, the scaffold functions as an active niche that supplies the cues a hostile host environment cannot. Injectable, self–healing hydrogels can establish a pro–angiogenic microenvironment for diabetic wounds by stabilizing HIF–1α [131], serve as oxygen reservoirs that meet the acute metabolic demand of neural grafts [132], or stage the sequential release of proliferative and differentiation–promoting growth factors to mirror injury biology [133]. Chemically defined matrices—macroporous hyaluronic–acid polyHIPEs and synthetic ultrashort–peptide hydrogels—further couple receptor–specific bioactivity with controlled architecture to support stem–cell maintenance and coordinated osteogenic–angiogenic output [134, 135]. The translational synthesis is that effective systems converge on a shared feature set—shear–thinning injectability, macroporosity, degradation synchronized to tissue deposition and defined composition—and should be designed as instructive niches rather than inert carriers.

#### 3.2.2 3D Bioprinting for Spatially Organized Constructs

3D bioprinting reframes adult stem cell therapy from a primarily cellular intervention into a problem of architectural design: the deterministic placement of stem cells, their derivatives, and supportive components into constructs that couple spatial patterning with microenvironmental control. The principle that should organize this section is that the value of bioprinting is realized only when the fabrication strategy is matched to the functional requirements of the target construct; printer modality and bioink are not interchangeable defaults but coupled design choices dictated by the resolution, cell density, construct size, vascularization need, and mechanical properties the application demands. That geometry itself can be instructive—additively manufactured lattices mechanotransductively program osteogenesis in adipose MSCs without biochemical inducers [136]—illustrates why these fabrication choices are not merely technical but determine biological outcome.

No single bioprinting modality is optimal across applications, and the principal selection criterion is the trade‐off between resolution, cell viability, throughput, and bioink compatibility summarized in Table 1. Extrusion‐based printing accommodates high cell densities and high‐viscosity, structurally robust bioinks, making it the default for large or load‐bearing constructs, but its resolution is limited, and nozzle shear can reduce viability. Droplet‐based (inkjet) and laser‐assisted printing achieve substantially finer resolution and higher viability—laser‐assisted printing approaching single‐cell placement—at the cost of throughput and a requirement for low‐viscosity inks. Light‐based methods (stereolithography and digital light processing) cure entire layers at once, combining fine internal architecture with comparatively fast build times, but require photocrosslinkable bioinks. The practical consequence is that modality selection should follow the construct specification: extrusion or coaxial printing for vascularized or structural tissue, where coaxial nozzles additionally enrich therapeutic small‐extracellular‐vesicle output for bone repair [137], and light‐ or laser‐based methods where geometric precision is the dominant requirement.

**TABLE 1:** Comparison of major 3D bioprinting modalities for adult stem cell construct fabrication.

Modality	Resolution	Cell viability	Speed/throughput	Compatible bioinks	Best‐suited application
Extrusion‐based	Moderate (∼100 µm)	Moderate; shear‐dependent	Moderate	Broad; high‐viscosity, high cell density	Large or load‐bearing constructs
Droplet / inkjet	High (∼20–50 µm)	High	High	Low‐viscosity inks only	Thin constructs; high‐throughput cell patterning
Laser‐assisted (LIFT)	Very high; near single‐cell	High	Low	Moderate viscosity; nozzle‐free	Precise cell placement; fine patterning
Light‐based (SLA / DLP)	High; fine internal detail	High	High; layer‐parallel	Photocrosslinkable inks (e.g., GelMA)	Complex internal architecture

The bioink is as decisive as the printer, and its central constraint is the well‐recognized trade‐off between printability and biological functionality—the “biofabrication window.” Inks stiff and fast‐gelling enough to hold a designed shape often constrain cell spreading, matrix remodelling and differentiation, whereas soft, cell‐permissive inks print with poor fidelity. State‐of‐the‐art bioinks therefore decouple these requirements—through shear‐thinning and rapidly stabilizing chemistries, composite or interpenetrating networks, and sacrificial supports—so that mechanical printability and post‐printing biological performance can be tuned independently. Because bioink mechanics are themselves instructive, they act as explicit levers of fate: tuning bioink stiffness and adhesivity steers lineage choice and construct fidelity in pancreatic progenitor constructs [138]. Processing parameters—crosslinking mode and kinetics, and post‐printing maturation or perfusion—are an equally important and frequently under‐specified determinant of whether a printed construct attains its intended properties.

**FIGURE 11:**
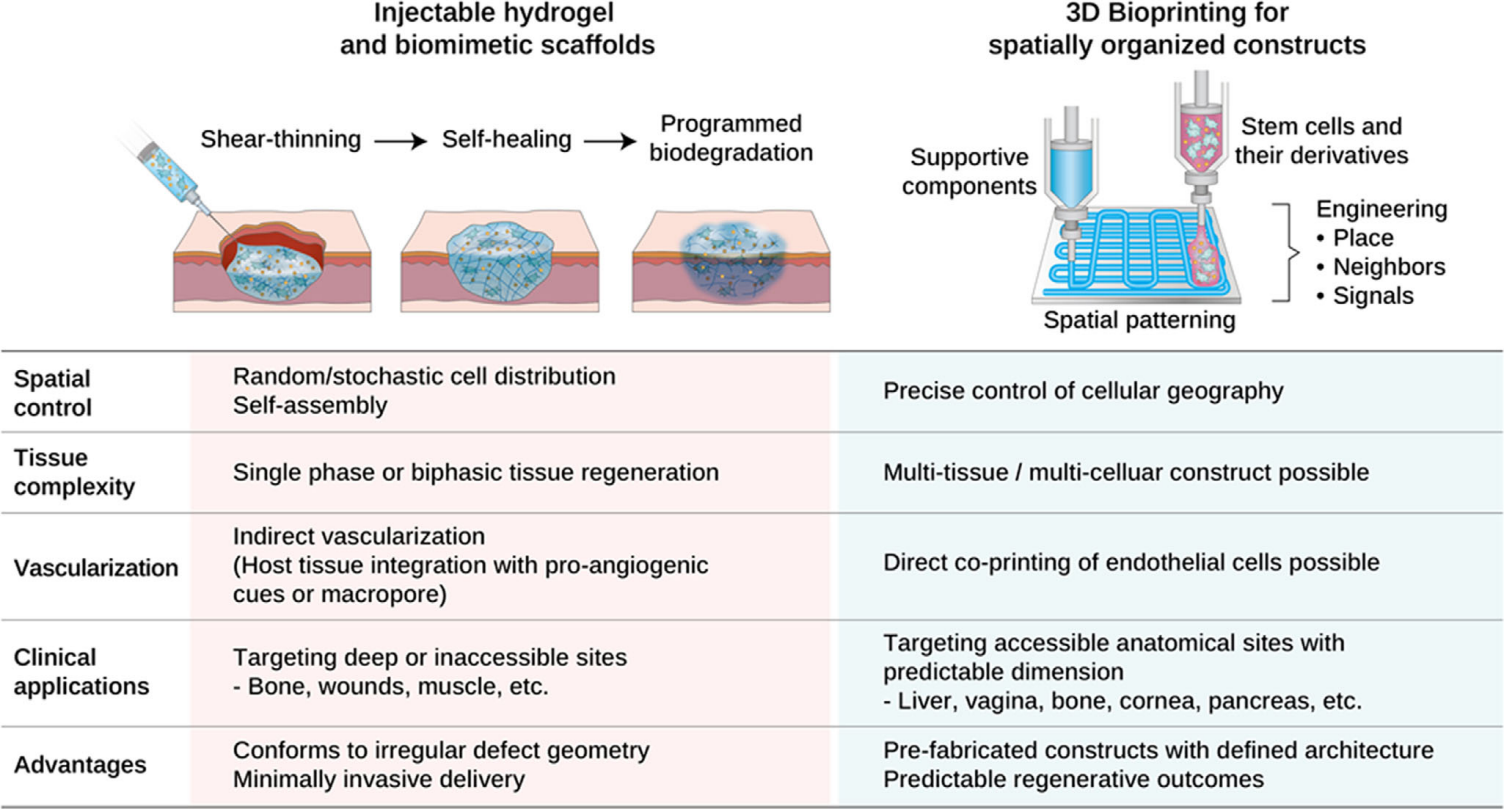
Comparison of bioengineering platforms to enhance stem‐cell therapy: injectable hydrogels/biomimetic scaffolds versus 3D bioprinting. Injectable hydrogels and biomimetic scaffolds (left) provide a deployable niche‐design toolkit that enables minimally invasive delivery while protecting transplanted cells and programming their behavior through material properties. Representative features include shear‐thinning injectability, rapid self‐healing after delivery, and programmed biodegradation that supports cell–matrix integration and tissue replacement. In contrast, 3D bioprinting for spatially organized constructs (right) enables deterministic control over cellular geography by patterning stem cells (and derivatives) together with supportive components, thereby engineering “place, neighbors, and signals” within an architected microenvironment. The accompanying table summarizes key trade‐offs between the two approaches in terms of spatial control (stochastic/self‐assembled distribution vs precise patterning), achievable tissue complexity (single/biphasic regeneration vs multi‐tissue/multicellular constructs), vascularization strategy (indirect host‐driven ingrowth vs potential for direct co‐printing of endothelial/support cells), clinical targeting (deep/inaccessible sites vs anatomically accessible sites with defined dimensions), and practical advantages (conformability to irregular defects vs prefabricated architecture with predictable outcomes). Original figure created by the authors. Copyright 2026, The Authors.

Spatially organized printing has now translated into coherent functional gains across hepatic, vaginal, corneal and neural constructs—through hepatic microtissue sheets [139], exosome‐laden lumen‐mimetic scaffolds [26], multi‐material corneal stromal lamellae [140] and directionally patterned neural‐progenitor circuits [28]—supporting bioprinting as a route to standardize the host context an adult stem cell will encounter. The principal barrier to translation is no longer feasibility but scalability: clinically relevant constructs demand build volumes, print speeds and batch‐to‐batch reproducibility well beyond typical laboratory demonstrations, together with clinical‐grade, consistent bioinks and processes compatible with good manufacturing practice. Refocusing bioprinting development around these scalability and standardization requirements—rather than around an ever‐widening catalogue of tissue demonstrations—is, in our view, the step most likely to convert architected constructs into deliverable adult stem cell therapies. As summarized in Figure 11, injectable hydrogels/biomimetic scaffolds and 3D bioprinting represent complementary bioengineering strategies to enhance stem–cell therapy, differing in spatial control, construct complexity, vascularization potential, and translational application.

### 3.3 Genetic and Epigenetic Modifications

#### 3.3.1 Overexpression of Pro‐Survival and Pro‐Regenerative Genes

Genetic augmentation of adult stem cells reframes cell therapy as the rational installation of survival, immunoregulatory, and tissue‐repair programs. Rather than relying on hostile host niches to supply pro‐repair cues, overexpression strategies position MSCs as active bioreactors: secreting trophic factors, resisting oxidative and apoptotic stress, modulating innate–adaptive immunity, and, when needed, acquiring lesion‐matched trafficking or lineage‐priming capacities. Across diverse contexts, enforced expression of secreted effectors (e.g., angiogenic and endocrine mediators), master transcriptional regulators that rejuvenate senescent stem cells, innate‐sensing nodes that polarize graft immunity, and chemokine receptors that align homing with disease chemotactic fields has yielded convergent gains—superior engraftment quality, durable paracrine potency, balanced Treg/Th17 and pro‐/anti‐inflammatory cytokine profiles, enhanced mitochondrial/energetic fitness, and accelerated tissue reconstruction.

Genetic augmentation reframes cell therapy as the rational installation of survival, immunoregulatory, and tissue–repair programs, positioning MSCs as engineered bioreactors rather than passive grafts dependent on a hostile host niche. Across diverse disease contexts, enforced expression of secreted effectors, master transcriptional regulators and homing receptors yields convergent gains in engraftment quality, paracrine potency, immune balance and tissue reconstruction. Secreted–factor and endocrine programs illustrate the approach: PEDF overexpression restores ovarian function by expanding regulatory T cells and normalizing hormone profiles [20], human growth hormone accelerates burn repair through ERK–dependent signaling [21], and Nanog confers a pro–survival, pro–regenerative phenotype with improved mitochondrial fitness and amplified exosome biogenesis after myocardial infarction [22].

**FIGURE 12:**
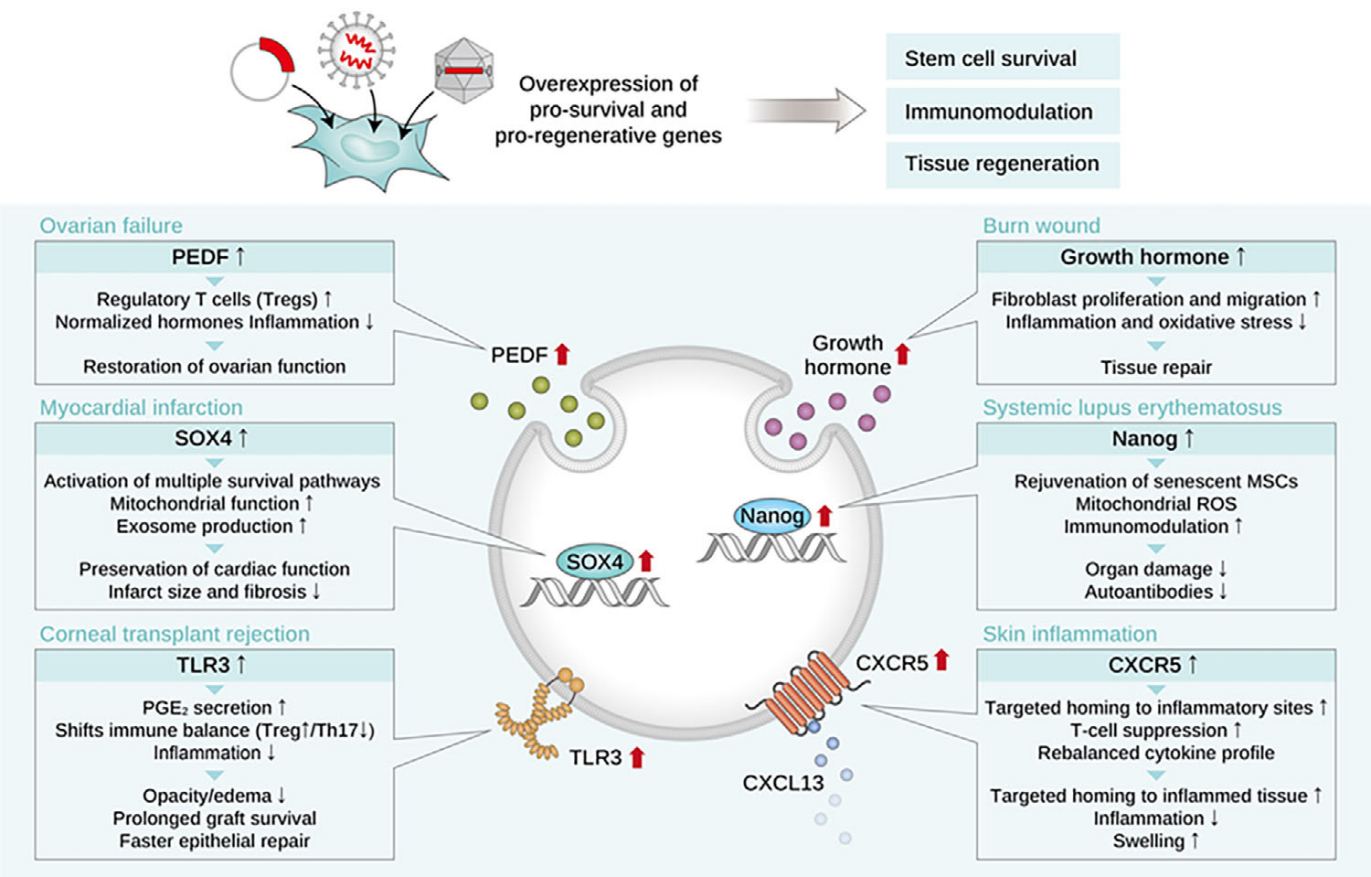
Genetic augmentation of MSCs by overexpression of pro‐survival and pro‐regenerative genes enhances therapeutic efficacy across diverse disease microenvironments. Schematic overview illustrating how genetic engineering approaches (e.g., viral or non‐integrating vector–mediated gene delivery) can “install” reparative programs in adult mesenchymal stem cells (MSCs), thereby reinforcing stem cell survival, immunomodulation, and tissue regeneration after transplantation. Representative examples include: (i) PEDF overexpression to restore ovarian function by increasing regulatory T cells (Tregs), normalizing hormone profiles, and suppressing inflammation; (ii) growth hormone (HGH) overexpression to accelerate burn wound repair by enhancing fibroblast proliferation/migration while reducing inflammatory and oxidative stress; (iii) Nanog overexpression to promote a pro‐survival, pro‐regenerative phenotype via improved mitochondrial fitness and amplified immunomodulatory capacity; (iv) SOX4 overexpression to rejuvenate senescent MSCs and strengthen immunoregulatory potency; (v) TLR3 overexpression to mitigate high‐risk corneal graft rejection by increasing PGE_2_ production, shifting immune balance toward Tregs (Treg↑/Th17↓), lowering inflammation, and improving epithelial repair/graft survival; and (vi) CXCR5 overexpression to enhance targeted homing toward CXCL13‐rich inflammatory sites, intensifying local T‐cell suppression and rebalancing cytokine profiles to reduce tissue inflammation. Original figure created by the authors. Copyright 2026, The Authors.

A second class of targets installs durable cell–state or trafficking changes: SOX4 overexpression rejuvenates senescent MSCs and restores immunomodulatory potency in lupus [141], TLR3 overexpression shifts graft immunity toward a tolerogenic Treg–high state in corneal transplantation [142], CXCR5 overexpression aligns homing with disease–specific CXCL13 chemotactic fields [143], and Myl2 reprograms brown adipose progenitors toward thermogenic adipocytes [144]. Critically, however, the literature also delivers an essential cautionary counterpoint that should temper an indiscriminate “more survival signaling is better” logic: enforced AKT1 expression accelerates replicative senescence and potentiates osteoclastogenic signaling, demonstrating that not every pro–survival gene is pro–regenerative [145], while hypoxic engagement of BMP–9 circuitry enhances osteogenic priming in vitro yet does not always translate to superior in vivo bone formation [146]. The translational synthesis is therefore that genetic augmentation is powerful but target–sensitive: the choice of transgene must be mechanism–anchored and matched to the specific disease microenvironment rather than assumed beneficial. Genetic augmentation strategies that enhance MSC survival, immunomodulation, and tissue–repair programs are summarized in Figure 12.

#### 3.3.2 Epigenetic Modulation of Regenerative Gene Networks

Epigenetic regulation—encompassing DNA methylation, histone post‐translational modifications, nucleosome remodeling, and enhancer topology—constitutes a decisive layer of control over adult stem‐cell fate and therapeutic performance. Across diverse models, targeted manipulation of this layer has proven sufficient to convert dysfunctional or senescent cells into regeneration‐competent states, to re‐establish lineage programs, and to stabilize reparative paracrine signaling. Recent observations frame epigenetic engineering as a powerful yet precision‐sensitive lever: the choice of writer/eraser/reader targets, dosing, and timing must be tuned to cell state and indication to avoid off‐target toxicity or maladaptive network states. In sum, rational epigenetic modulation transforms adult stem‐cell therapy from cell replacement into programmable network repair—reactivating latent regenerative circuits, stabilizing reparative phenotypes, and improving the durability and predictability of therapeutic benefit.

Epigenetic regulation—DNA methylation, histone modification, nucleosome remodeling and enhancer topology—constitutes a decisive and programmable layer of control over adult stem–cell fate. The principle emerging across diverse models is that targeted manipulation of this layer can convert dysfunctional or senescent cells into regeneration–competent states, reframing cell therapy as programmable network repair rather than simple cell replacement. Disease– and age–associated dysfunction is frequently an epigenetic, and therefore reversible, lesion: hyperglycemia represses osteogenic gene networks through chromatin changes that DNMT or HDAC inhibition can reopen [147], and ASXL2 acts as a PR–DUB/Polycomb rheostat whose loss silences osteogenic competence [148]. Histone–deacetylase inhibition similarly reactivates cardiomyogenic networks, most strongly in disease–derived MSCs [107].

**FIGURE 13:**
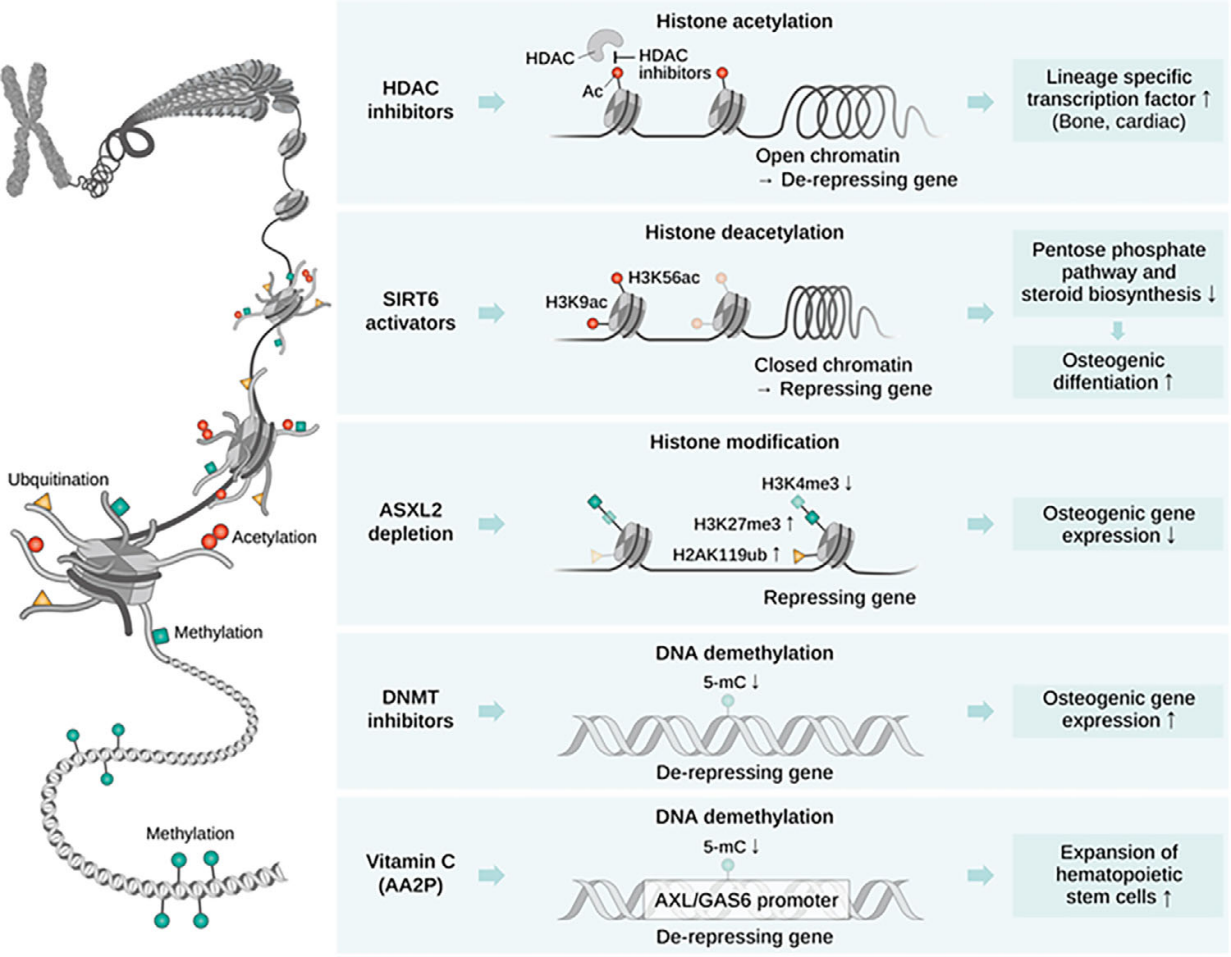
Epigenetic modulation strategies to reprogram adult stem/progenitor cells toward regeneration‐competent states. Schematic overview of how targeted manipulation of chromatin marks and DNA methylation converts “closed” (repressed) versus “open” (accessible) chromatin into tunable transcriptional outputs that control stem‐cell fate and therapeutic potency. Representative histone modifications (ubiquitination, acetylation, methylation) and CpG methylation (5‐mC) are shown as actionable regulatory layers. Pharmacologic HDAC inhibition increases histone acetylation, relaxes chromatin, and de‐represses lineage‐relevant gene programs to enhance differentiation/repair outcomes (e.g., osteogenic/cardiomyogenic activation). SIRT6 activation decreases H3K9ac/H3K56ac and rewires metabolic/lineage networks to promote osteogenic commitment. ASXL2 loss perturbs PR‐DUB/Polycomb balance (↑H2AK119ub, ↑H3K27me3, ↓H3K4me3), reinforcing transcriptional repression and weakening osteogenic gene expression. DNMT inhibition reduces DNA methylation (5‐mC) to re‐open silenced loci and restore osteogenic circuitry under pathological conditions such as hyperglycemia. Vitamin C derivative–driven TET‐dependent DNA hypomethylation de‐represses the GAS6–AXL axis, enabling robust ex vivo expansion and improved functional competence of hematopoietic stem/progenitor cells. Original figure created by the authors. Copyright 2026, The Authors.

The same logic enables proactive enhancement of regenerative capacity. SIRT6 activation rewires metabolic chromatin to drive ossific commitment and hard–tissue repair [16], Sirt6–enriched extracellular vesicles recalibrate stress–repair programs in ischemic myocardium [25], and TET–dependent DNA hypomethylation de–represses the GAS6–AXL axis to enable robust ex vivo expansion of hematopoietic stem/progenitor cells [149], while enhancer–level remodeling can reshape inflammatory gene programs in epithelial progenitors [150]. The decisive qualification for translation is that epigenetic engineering is precision–sensitive: the choice of writer, eraser, or reader target, together with dose and timing, must be matched to cell state and indication to avoid off–target toxicity or maladaptive network states. Used with this precision, rational epigenetic modulation reactivates latent regenerative circuits and improves the durability and predictability of therapeutic benefit. The key concepts and representative mechanisms of epigenetic modulation strategies to enhance adult stem/progenitor cell regenerative function are summarized in Figure 13.

## 4 Clinical Translation and Regulatory Perspectives

### 4.1 Current Clinical Trial Outcomes and Limitations

The translational landscape is advancing but uneven. Across early‐ and mid‐phase clinical studies, adult stem cell products (particularly MSCs) are consistently deliverable with acceptable safety and adherence, and several programs report signal‐level improvements in disease activity or tissue regeneration under rigorous monitoring [30, 31, 32, 33]. Still, the field remains methodologically fragile: many studies are small, single‐center, or single‐arm; sourcing, manufacturing, dose/schedule, and delivery routes vary widely; outcome measures and follow‐up horizons are heterogeneous; and correlative biomarker frameworks (e.g., MRD, imaging, or fluid biomarkers) are often under‐specified. These realities complicate meta‐analysis, payer deliberations, and the development of robust patient‐selection rules—highlighting the need for adequately powered, multicenter randomized trials built on harmonized manufacturing controls, potency assays linked to mechanism, and standardized clinical/biomarker endpoints.

Across contemporary trials, adult stem cell–based interventions are consistently deliverable and safe at the point of care, with multiple programs reporting signal‐level clinical improvements in disease activity or tissue repair under rigorous monitoring. Feasibility spans systemic and local delivery paradigms—from repeated intravenous dosing of allogeneic products in neurodegeneration and systemic autoimmunity, to minimally invasive, chairside injections for focal regenerative indications—while standard peri‐procedural workflows can be preserved. Early randomized experiences further suggest that structured trial conduct and masking are achievable and that patient adherence is acceptable, supporting the translational viability of adult stem cell therapeutics in routine clinical settings.

Chen et al. reported that contemporary clinical trials of adult stem cell therapies for autoimmune diseases show consistent signals of feasibility and short‐term disease control, particularly with MSC products, while durability and comparative efficacy remain unresolved due to the field's predominance of early‐phase, single‐arm studies [151]. Outcomes across indications are heterogeneous and appear to depend on disease biology and delivery strategy (e.g., systemic infusion for systemic autoimmunity versus local administration for focal lesions), with MSCs constituting the dominant cell source and allogeneic products and intravenous routes most commonly used. Safety profiles are generally acceptable for MSC‐based interventions, whereas hematopoietic stem cell–based regimens carry a higher burden of serious treatment‐related adverse events, reflecting the intensity of conditioning approaches. However, clinical interpretation is constrained by limited late‐phase trials, geographic concentration of research sites, and methodological heterogeneity in cell sourcing, manufacturing, dosing, endpoints, and follow‐up, compounded by high costs and insufficient long‐term safety data [151].

Fernandes et al. reported that intravenous allogeneic human dental pulp stem cell therapy (NestaCell) for Huntington's disease was clinically feasible, well tolerated, and produced signal‐level improvements on prespecified motor and functional endpoints in a randomized, double‐blind, placebo‐controlled phase II setting—supporting the deliverability of repeated systemic dosing over ∼1 year with an acceptable safety profile and no treatment‐related serious adverse events [152]. They. also underscored key limitations that temper inference: a single‐center, small‐sample early‐phase design; baseline imbalances across arms; imaging constraints (chorea‐related motion artifacts limiting MRI analyses); and incomplete biomarker incorporation (e.g., absence of neurofilament light chain), all of which restrict generalizability and mechanistic insight. The authors conclude that while short‐term outcomes are encouraging, confirmatory multicenter trials with harmonized endpoints, strengthened imaging/biomarker readouts, and longer follow‐up are required to establish durability of benefit and define optimal candidates and dosing [152].

El‐Jawahri et al. reported that a clinician‐delivered, multimodal sexual health intervention for survivors of haematopoietic stem‐cell transplantation was clinically feasible, acceptable, and efficacious in a randomized comparison against enhanced usual care, yielding broad improvements across patient‐reported sexual function, quality of life, and psychological distress without new safety signals. The program leveraged brief training of existing transplant clinicians and was deliverable in person or via telehealth—features that underscore scalability and pragmatic integration into survivorship care pathways [32]. They also highlighted limitations typical of early supportive‐care trials: single‐centre enrollment with limited sociodemographic diversity, open‐label design with reliance on patient‐reported outcomes, short follow‐up that precludes conclusions about durability, and restricted power for subgroup analyses by transplant type and sex. Potential contamination from shared clinicians and the absence of partner‐level outcomes further temper generalizability [32].

Liu et al. reported that allogeneic dental pulp stem cell (DPSC) injection for chronic periodontitis is clinically feasible, well tolerated, and operationally scalable as a minimally invasive, chairside adjunct to standard debridement. In two randomized, placebo‐controlled trials across two centers, DPSC therapy showed consistent safety without serious adverse events and signal‐level improvements across both soft‐ and hard‐tissue endpoints (attachment loss, probing depth, bone defect depth), with more pronounced benefits in stage III disease—supporting deliverability of a locally injected adult stem‐cell product in real‐world periodontal care [153]. They also underscored key limitations that temper efficacy inference: short 6‐month follow‐up, small per‐arm sample sizes, and reliance on post hoc subgroup analyses (e.g., disease stage, root anatomy, defect morphology) that introduce multiplicity and selection bias. Heterogeneity in dosing schedules (single vs. double injection) and tooth‐level analyses further constrains generalizability, and the trials were limited to two centers. Optimal dose, retreatment interval, and patient selection remain undefined, and durability beyond six months is unknown [153].

Schulman et al. reported that peri‐grafting application of allogeneic BM‐MSCs to a deep second‐degree burn was clinically feasible, well tolerated, and operationally compatible with standard surgical workflows—yielding qualitative improvements in wound‐bed granulation, earlier readiness for split‐thickness skin grafting, and downstream gains in graft appearance and sensory recovery without signs of rejection or new safety signals [154]. They also emphasized limitations that temper inference: the evidence derives from a single case embedded within a phase‐I framework (and ultimately excluded from the parent trial), lacks a concurrent control, and relies predominantly on clinical observation and patient‐reported scar scales over short‐term follow‐up—factors that constrain generalizability and preclude conclusions about durability or dose/route optimization [154].

Kawamura et al. reported that initiating lenalidomide maintenance well after allogeneic transplantation of hematopoietic stem cells was clinically feasible, with manageable toxicity and acceptable treatment adherence, and it showed signals of disease control in a subset of patients. Safety monitoring indicated no unexpected immunologic complications in the early maintenance window, and limited correlative assays suggested stability of major lymphocyte compartments during therapy. Overall, the trial supports the deliverability of a delayed‐start maintenance approach in a high‐risk, post‐transplant setting [155]. They also emphasized substantial limitations that temper interpretation of benefit. The study was single‐arm with a small, heterogeneous cohort and broad variability in maintenance start times, leaving efficacy underpowered and susceptible to selection bias. The planned expansion phase was not completed, and several key correlative endpoints (including standardized MRD assessments) were incomplete, constraining mechanistic insight.

Notwithstanding these advances, the evidentiary base remains methodologically fragile. The field is dominated by early‐phase, often single‐center and small‐sample studies, with heterogeneous cell sources, manufacturing specifications, dosing schedules, routes of administration, and response assessments that complicate cross‐study comparison. Follow‐up is frequently short, limiting conclusions about durability, retreatment cadence, and long‐term safety. Many trials lack standardized biomarker frameworks (e.g., MRD, molecular imaging, or fluid biomarkers), curtailing mechanistic inference and patient stratification. Practical constraints—such as baseline imbalances, imaging artifacts in movement disorders, and reliance on patient‐reported outcomes—introduce additional interpretability challenges. Collectively, current trials establish clinical feasibility and early benefit signals, but they also delineate a clear agenda: multicenter, adequately powered randomized studies with harmonized manufacturing and endpoints, integrated biomarker readouts, longer surveillance, and prospectively defined selection criteria to resolve durability, optimize dosing and delivery, and credibly quantify comparative efficacy.

### 4.2 Global Regulatory Frameworks and Standardization Issues

Regulatory architecture and standardization further shape what “optimized” can credibly reach patients. Major jurisdictions classify adult stem cell products within risk‐based medicinal categories and require GxP‐anchored manufacturing, pharmacovigilance, and CTD‐style dossiers, but key definitions (minimal vs. substantial manipulation, homologous use), GMP/GTP interpretations, potency/identity assay expectations, and registry transparency are not fully aligned across borders [34, 35, 36]. Dual‐track systems that encourage early clinical exploration have accelerated pipelines yet exposed gaps in quality systems and data portability from investigator‐initiated studies to registration‐grade evidence. In parallel, the visibility afforded by trial registries can be co‐opted by unregulated offerings, necessitating clearer disclosure of competent‐authority oversight and IRB review and stronger inspection/enforcement. Looking forward, frameworks must also anticipate new modalities—engineered exosomes, organoid‐derived products, and gene‐edited cells—to ensure that ASC therapies advance under harmonized, enforceable, and evidence‐generating rules.

The rapid global expansion of adult stem‐cell trials has outpaced the consolidation of a coherent regulatory architecture. Jurisdictions classify and govern these products through divergent, risk‐based pathways (e.g., advanced therapies, biologics, regenerative medical products), yielding non‐aligned evidentiary thresholds, uneven approval portfolios, and limited cross‐border interoperability. In parallel, dual‐track systems that encourage early clinical exploration (e.g., investigator‐initiated studies) have accelerated pipeline growth but exposed gaps in quality systems, manufacturing consistency, and data portability from exploratory studies to registration‐grade evidence. Trial registries have increased transparency, yet registration alone does not equate to competent authority oversight, enabling opportunistic markets—including direct‐to‐consumer “secretome/exosome” offerings and stem‐cell tourism—to appropriate the language of clinical research.

Li et al. reported that the global clinical trial ecosystem for adult stem cell therapies has expanded rapidly but remains regulatory‐fragmented and methodologically heterogeneous, complicating aggregation of evidence and cross‐border oversight. Trial registries have broadened visibility, yet registration does not imply supervision by a competent drug authority, and the same infrastructure gaps that enable legitimate early‐phase work also permit unregulated clinics and “stem‐cell tourism” to appropriate the rhetoric of clinical research. The authors argue for greater transparency in registries—including explicit identification of the overseeing regulatory body and research ethics board—and for harmonized expectations around trial design, endpoints, and follow‐up to align claims with clinical realities [156]. They also highlighted standardization deficits across the development pipeline: inconsistent sourcing and phenotyping, variable manufacturing controls/GMP access, heterogeneous dosing and delivery routes, and nonuniform response measures that hinder comparability and reimbursement decisions. They note the rise of allogeneic “off‐the‐shelf” MSC products alongside point‐of‐care autologous devices, each posing distinct regulatory and immunological challenges that demand convergent quality systems, potency assays, and safety monitoring [156].

Wong et al. reported that national oversight of adult stem‐cell therapies remains fragmented and guidance‐driven rather than statute‐anchored in many jurisdictions, exemplified by Malaysia's reliance on Ministry of Health and NPRA guidelines (e.g., HSCT standards, cord‐blood banking, and CGTP registration) in the absence of a dedicated stem‐cell law. Within this framework, only umbilical‐cord and hematopoietic stem‐cell transplantation are permitted as standard care, whereas other somatic and embryonic stem‐cell uses are restricted to the experimental domain and xenotransplantation is prohibited [34]. The authors identify systemic standardization gaps—inconsistent sourcing and characterization, variable manufacturing and quality systems, heterogeneous dosing and delivery, and nonuniform outcome measures—that hinder comparability, reimbursement decisions, and global evidence synthesis. They further highlight emergent risks—the proliferation of unregulated clinics, direct‐to‐consumer “secretome/exosome” offerings, and stem‐cell tourism—amplified by uneven enforcement and limited public literacy. To close these gaps, the commentary calls for specific legislation tailored to cell therapies; strengthened inspection and enforcement; risk‐based CGTP registration with Good Tissue Practice; alignment with international norms (e.g., ISSCR guidance); and updates to cover new modalities (organoids, gene editing) [34].

Du et al. reported that major regulators have established dedicated pathways for cell therapies (FDA/EMA/PMDA), while China's dual‐track framework—introduced in 2017 to separately enable clinical research and product registration—has catalyzed rapid pipeline growth to the world's second largest. Despite this expansion, the authors highlight global standardization gaps, including redundant R&D and target clustering, and emphasize that approval portfolios remain uneven across regions relative to disease burden, underscoring the need for harmonized expectations spanning indications, targets, and evidentiary thresholds [35]. They further noted that China's landscape is dominated by investigator‐initiated trials (IITs), which, while flexible and prolific, raise quality‐system and manufacturing consistency challenges that limit the translational value of early signals. To strengthen regulatory science and reduce duplication, the authors recommend: (i) stricter standards to curb redundant within‐class programs; (ii) companion‐diagnostic co‐development (as encouraged by FDA guidance) to sharpen patient selection; (iii) value‐based reimbursement to disincentivize me‐too products; and (iv) clearly specified technical requirements for using IIT data in IND applications, creating a predictable pathway from exploratory studies to registration‐grade evidence [35].

López‐Beas et al. reported that major regulators classify MSC products as medicinal products within distinct, risk‐based frameworks—ATMPs in the EU, HCT/Ps (biologics) under FDA/CBER in the United States, regenerative medical products in Japan (PMDA), and biologicals/biologics in Australia (TGA), Canada (Health Canada), and South Korea (MFDS)—with convergent requirements spanning clinical‐trial authorization of investigational products (IMPs), GMP/GTP‐anchored manufacturing and distribution, pharmacovigilance, and dossier submission in CTD format for marketing authorization. Compassionate‐use pathways exist but remain tightly controlled. Despite a large trial portfolio, only a small number of MSC products had achieved approval worldwide at the time of review, underscoring the translational bottleneck between early clinical experience and licensure [36]. They. further highlighted persistent standardization gaps that complicate cross‐study comparability and global convergence: jurisdiction‐specific definitions of minimal vs. substantial manipulation and homologous use; heterogeneous cell sourcing and phenotyping; variability in potency and identity assays (despite ISCT minimal criteria); inconsistent dosing, routes, and regimen design; and the need for comparability plans when manufacturing processes evolve [36]. The authors also note divergent GMP interpretations (e.g., EMA ATMP‐specific GMP, FDA cGTP) and emphasize harmonized nonclinical and clinical expectations (ICH S6(R1), EMA/FDA guidance) to align risk–benefit evaluations. Finally, they warn that registry visibility without robust oversight can enable unregulated offerings and “stem‐cell tourism,” arguing for clearer registry transparency (regulatory authority and IRB identification), unified technical standards, and multicenter randomized trials built on shared endpoints to support credible efficacy claims and equitable patient access.

Standardization deficits permeate the development lifecycle. Cell sourcing and phenotyping remain heterogeneous; definitions of minimal versus substantial manipulation and homologous use vary across regulators; GMP/GTP interpretations diverge; and potency/identity assays lack universal, mechanism‐anchored benchmarks. Dosing schemes, routes of administration, and regimen design are inconsistent, as are clinical endpoints and follow‐up windows, complicating meta‐analysis, reimbursement deliberations, and post‐market pharmacovigilance. As manufacturing evolves, formal comparability plans are often under‐specified, impeding continuous process improvement without resetting clinical evidence. Addressing these gaps requires regulatory convergence on technical standards and dossier expectations, explicit registry disclosures (oversight authority and ethics board identification), risk‐based inspection and enforcement, and integration of biomarker frameworks and companion diagnostics to sharpen patient selection. Finally, frameworks must anticipate new modalities—such as organoids, engineered exosomes, and gene‐edited products—so that adult stem‐cell interventions advance under harmonized, enforceable, and evidence‐generating rules that support credible efficacy claims and equitable access.

## 5 Conclusion and Future Perspectives

Durable clinical benefit from adult stem‐cell therapies will be realized when “cells” and “context” are co‐designed: the right molecular programs must be installed in cells and deployed within spatially defined, instructive microenvironments under clear translational rules. This systems view highlights three interlocking levers—cell‐intrinsic programs (source, transcriptional/epigenetic state, senescence, and metabolic fitness), microenvironmental constraints and designable cues, and engineering interventions applied pre‐ and post‐delivery—that together explain why in vitro potency only partly predicts in vivo performance. Advances in spatially organized biofabrication, particularly 3D bioprinting that tunes geometry, stiffness, and factor presentation, further recast cell therapy from simple infusion into an architected niche that stabilizes survival, aligns angiogenesis with tissue architecture, and accelerates integration.

Translationally, early‐ and mid‐phase trials indicate that adult stem‐cell products—especially MSCs—are broadly deliverable with acceptable safety, yet the evidence base remains methodologically fragile due to small, single‐center or single‐arm designs; heterogeneous sourcing, manufacturing, dose/schedule, and delivery; and non‐standard endpoints and biomarker frameworks. The field should therefore prioritize adequately powered, multicenter randomized trials conducted under harmonized manufacturing controls and mechanism‐linked potency assays, with standardized clinical and biomarker endpoints to enable rigorous meta‐analysis and robust patient selection.

Regulatory architecture will ultimately shape what “optimized” can credibly reach patients. Convergence on technical standards—definitions of manipulation and homologous use, GMP/GTP interpretations, potency/identity assay expectations, formal comparability plans—as well as explicit registry transparency and risk‐based inspection/enforcement is essential to curb unregulated offerings and to create predictable pathways from exploratory studies to registration‐grade evidence. Companion diagnostics and biomarker frameworks should be operationalized to sharpen patient selection and reduce redundant within‐class programs.

Looking ahead, the most credible roadmap is integrative: (i) install pro‐survival and pro‐regenerative circuits with genetic/epigenetic precision; (ii) embed cells within diffusion‐aware, geometry‐informed, and factor‐programmable constructs to standardize host context; and (iii) prosecute trials under convergent, enforceable, and explicitly evidence‐generating rules. Pursued together, these strategies provide a practical route from bench potency to durable, reproducible patient benefit. Finally, because MSCs have served as the principal exemplar throughout this review, a key priority for future work is the systematic validation of these determinants and engineered interventions across other adult stem cell populations, for which the present cells–context–interventions framework offers a structured starting point.

## Author Contributions

**S.R.K**., **Y.J.J**., organized manuscript, and wrote the paper. **H.Y.L**. designed manuscript, wrote the paper, and revised manuscript.

## Conflicts of Interest

The authors have no competing interests as defined by *Advanced Science* or other interests that might be perceived to influence the results and/or discussion reported in this article.

## Data Availability

All data needed to evaluate the conclusions in the paper are present in the paper and/or the Supplementary Materials. Source data can be made available to collaborators upon reasonable request.
